# Big breakfast diet composition impacts on appetite control and gut health: a randomised weight loss trial in adults with overweight or obesity

**DOI:** 10.1017/S000711452610645X

**Published:** 2026-06-14

**Authors:** Claire Fyfe, Gillian Donachie, Petra Louis, Graham W. Horgan, Claus Mayer, Leonie Ruddick-Collins, Freda Farquharson, Alan W. Walker, Alexandra M. Johnstone

**Affiliations:** 1 The Rowett Institute, University of Aberdeen, Foresterhill, Aberdeen, Scotland AB25 2ZD, UK; 2 Biomathematics and Statistics Scotland, Foresterhill, Aberdeen, Scotland AB25 2ZD, UK

**Keywords:** Obesity, Appetite regulation, Breakfast, Energy balance, Fibre, Gut health, Protein-induced satiety

## Abstract

Growing evidence supports early eating to control appetite and energy balance but there are few controlled studies to assess the amount and/or type of breakfast meal. This randomised, within-participant, diet intervention examined the effects of higher-fibre (HF) and higher-protein (HP) breakfasts in adults with overweight/obesity. Nineteen healthy adults consumed two randomised 28-d weight loss (WL) diets, as higher-fibre (HFWL) or higher protein (HPWL), with all food provided. Both WL diets were designed as 45 %, 35 % and 20 % of calories to be consumed in the morning, afternoon and evening, respectively. The primary outcome was energy balance, analysed by body weight changes. The secondary outcomes were gut health (assessed by changes in faecal microbiota composition and microbial metabolite concentrations) and subjective appetite assessed with visual analogue scales. There was a diet effect on WL, with mean loss of −4·87 kg and −3·87 kg for the HFWL and HPWL diets, respectively (*P* = 0·002). The HPWL diet was superior to the HFWL diet for suppressing subjective appetite (*P* = 0·003). The faecal microbiota analysis showed beneficial groups of bacteria, including bifidobacteria, and the butyrate producers *Faecalibacterium* and *Roseburia*, were significantly increased in proportional abundance on the HFWL diet. Breakfast composition has an important role in influencing subjective appetite with the HP diet promoting greater feelings of satiety. The proportional abundance of putatively beneficial groups of gut microbiota was markedly higher on the fibre-enriched diet, which may be preferable for gut health.

In addition to ‘what we eat’, there is evidence of the importance of ‘when we eat’ in the management of healthy weight. Recent diet intervention studies have challenged the assumption that a ‘calorie is a calorie’ in the context of energy metabolism for weight loss (WL)^(1,2)^, where the timing of meals, particularly when consumed in the morning period, may influence appetite control and metabolism. Garaulet *et al.* ^(3)^ reported that early eaters lost significantly more weight compared with late lunch eaters. Similarly, Jakubowicz *et al.* ^(4)^ reported that morning energy consumption resulted in greater WL, relative to evening energy consumption. It is known that morning energy consumption is associated with improved control of blood glucose, as well as lower hunger scores^(5,6)^, relative to evening eating, which may be due to a circadian effect of hormones and metabolism^(7)^ and/or a behavioural effect^(8)^. We have previously reported^(9)^ that the timing of daily energy loading did not influence energy metabolism or WL, but that appetite control was enhanced with a bigger breakfast meal. Further, Vujovic *et al* ^(10)^ reported that late eating increases hunger and promotes fat storage. This combined evidence supports regular breakfast consumption for body weight control, in tandem with avoidance of late eating. Despite strong public health advice on the importance of breakfast as part of a dietary approach for a healthy weight, very little is known about the importance of what is eaten in the morning period. Data are sparse on how and why time of meals, energy distribution and diet composition throughout the day relate to appetite control. Further, the impact of diet composition on markers of gut health has not been monitored in these controlled diet studies. Shifts in diet composition can result in a change of gut microbiota profile, often within days^(11,12)^. A high-protein ketogenic diet, being very low in carbohydrate and dietary fibre, dramatically decreases the health protective production of organic compounds, SCFA, produced by gut bacteria when they ferment dietary fibres. Specifically, of interest is butyrate, suggesting potential long-term undesirable impacts on gut health^(13)^. Conversely, optimal amounts of dietary fibre can contribute to a diverse microbiota profile and metabolites that are beneficial to health^(14)^. A large breakfast meal, high in fibre, could also usefully contribute to adults’ fibre dietary intake, which is commonly not met in westernised countries, leading to the ‘fibre gap’ (the gap between population dietary recommendations and intake)^(15)^.

Macronutrient composition of the diet has been shown to influence hunger and satiety^(16)^. Several studies have shown that dietary protein is the most satiating of the macronutrients in conditions of both energy restriction and energy balance^(17–19)^. Other work has highlighted the role of a big breakfast rich in protein and fat on improving glycaemic control^(20)^. High-protein meals are more satiating in the morning, reduce cravings and are more rewarding,^(21)^ and this may also be linked to sensory cues and texture of food/beverages consumed at this time of day. This is an apparent paradox since, in western culture, dinner is traditionally the largest meal of the day (40 % calories), with a smaller breakfast^(22,23)^. The mechanistic basis of appetite control of macronutrient loads at various times of the day has not been elucidated. This is important, with growing academic interest in the ‘real-world’ translation of circadian biology and nutrition (chrono-nutrition)^(7,8,24,25)^.

In summary, this work investigated the impact of a big breakfast meal (combined with a smaller evening meal) on energy balance, gut health and appetite. We hypothesised that morning eating, particularly a higher-protein (HP) meal compared with a higher-fibre (HF) meal, would be more satiating. Further, this may be linked to improved gastric emptying (GE) during the morning and activates enhanced downstream satiety mechanisms^(26,27)^.

## Participants and methods

**Figure 1.**
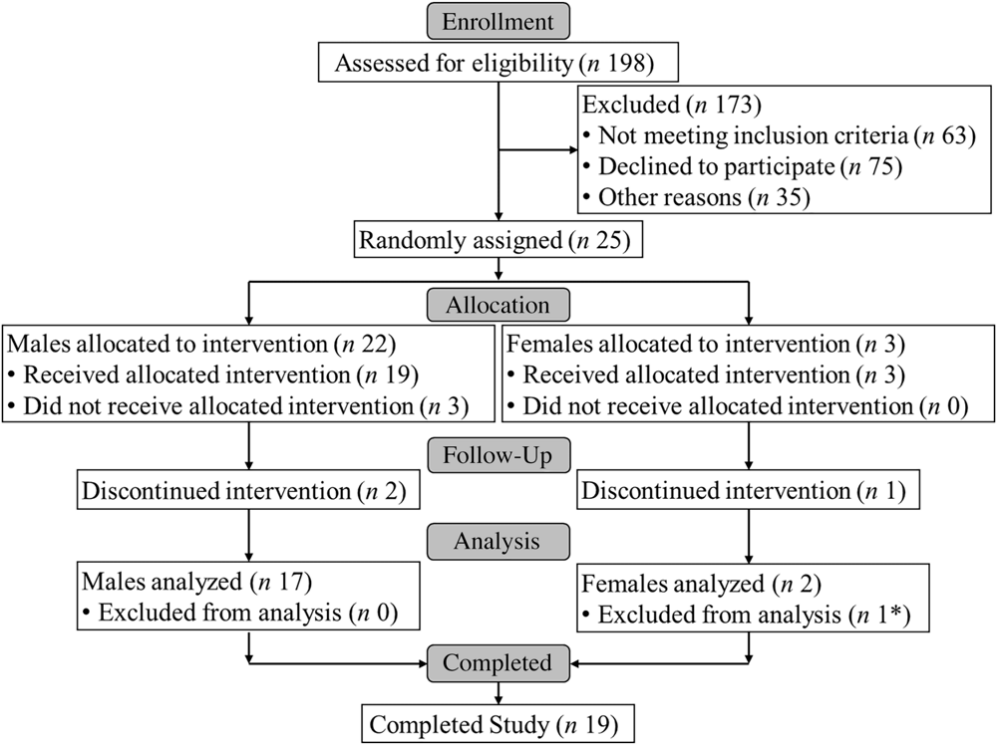
Figure 1 long description. CONSORT diagram summarising participant flow with the sizes (*n*) of initial (enrolled, recruited) and final groups. *One female participant was unable to provide a complete set of faecal samples so was not included in the analysis of faecal microbiota and microbial metabolites (*n* 18). Abbreviations: CONSORT, Consolidated Standards of Reporting Trials.

Recruitment was by public advertisement of a diet study for healthy, overweight or obese participants (BMI 27–42 kg/m^2^), aged 18–75 years. Inclusion criteria specified that all participants should be habitual breakfast eaters and not have existing medical conditions or take medication that could influence their appetite or mood. This was confirmed during the recruitment process by a medical screening questionnaire. Female participants were included if they were on hormonal contraceptives or were postmenopausal. Of the 198 people who expressed an interest in the study between March 2017 and April 2019, 2twenty-five were invited to attend a screening visit and to enter the study. All participants gave written, informed consent, after which they were randomly allocated to the diet sequence. After screening and allocation of intervention, three participants declined to take part and three participants had to withdraw for personal reasons; therefore, nineteen participants completed the study (two females and seventeen males), and their data are presented throughout. We were unable to collect a complete set of faecal samples from one participant, so all faecal microbiota and microbial metabolites analyses are presented for *n* 18. See Consolidated Standards of Reporting Trials flow diagram (Figure 1) summarising the participant flow. The study was conducted according to the ethics guidelines laid down in the Declaration of Helsinki^(28)^ and approved by the Rowett Ethics Review panel. The study is registered at CT.gov.UK under number NCT03077295. The CONSORT checklist for the study is in online supplementary material (online Supplementary Table 1).

**Figure 2.**
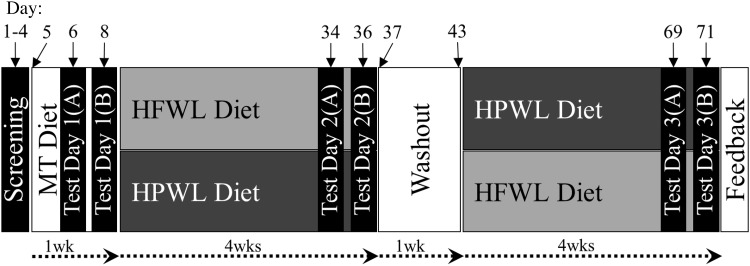
Experimental design for overweight adults (*n* 19)^1^ in a 71-d dietary protocol. ^1^Seventeen males and two females, age 38–72 years, BMI 27–41 kg/m^2^. Abbreviations: HFWL, High-fibre weight loss diet; HPWL, High-protein weight loss diet; MT, Maintenance diet.

All the study visits were conducted at the Human Intervention Studies Unit at the Rowett Institute (Aberdeen, UK). Dietetic staff of the Human Intervention Studies Unit supplied all food and drink consumed during the dietary periods, and participants attended three times per week for body weight assessment and food provision. The 71-d protocol was a randomised within-participant cross-over design (Figure 2) as follows: Days 1–4, *ad libitum* diet (4 d); Days 5–8, maintenance diet (MT, 4 d); Days 9–36 and 44–71, randomised HF weight loss diet (HFWL, 28 d) or HP WL diet (HPWL, 28 d). Days 37–43 were a 7-d washout period between the two WL diets, where the diet was the same composition as the MT. Fasting and postprandial measurements were conducted in the final week of each dietary period, across two test days. The baseline characteristics of the study participants recorded at screening and after the first MT dietary period are summarised in Table 1.

**Table 1.** Baseline anthropometric characteristics for the nineteen overweight and obese adults who completed the 71-d dietary protocol Table 1 long description.

	Mean	sem	Min	Max
Age (years)	57·4	1·90	38·0	72·0
Height (m)	1·75	0·01	1·64	1·89
Weight (kg)	102	3·50	78·7	139
BMI (kg/m^2^)	33·3	1·10	26·8	41·1
SBP (mmHg)	139	4·00	112	177
DBP (mmHg)	88·0	2·00	74·0	99·0
Measured RMR (kJ/d)^*^	8450	194	6790	12 200
Measured RMR (kcal/d)^*^	2020	46·0	1620	2930
Waist circumference (cm)	111	2·56	96·1	138
Hip circumference (cm)	111	2·22	101	140
WHR	1·01	0·02	0·84	1·17
Cholesterol (mmol/l)	5·48	0·36	2·10	8·22
Glucose (mmol/l)	5·97	0·24	4·93	9·71
HDL-cholesterol (mmol/l)	1·12	0·06	0·67	1·63
LDL-cholesterol (mmol/l)	3·38	0·29	1·04	5·57
NEFA (mmol/l)	0·61	0·04	0·28	0·90
TAG (mmol/l)	2·07	0·24	0·53	4·90
TC-HDL-cholesterol ratio	5·06	0·39	2·53	8·29
LDL-HDL-cholesterol ratio	3·11	0·28	1·25	5·87

Abbreviations: SBP, Systolic blood pressure; DBP, Diastolic blood pressure; WHR, Waist–hip ratio; TC, Total cholesterol.

* Measured by QUARK ventilated hood system.

### Formulation and preparation of the study diets

Participants were fed based on their measured resting energy requirements. RMR was measured after 30 min sat at rest using indirect calorimetry with a ventilated hood system (Quark RMR, COSMED) during the screening visit to determine MT diet energy requirements. This was used to scale diets to individual energy requirements. Measures at the end of MT allowed for recalculation of energy requirements for the following diet periods. The MT diet consisted of standard UK diet composition: 30 % fat, 15 % protein and 55 % carbohydrate fed to 1·5 × measured RMR as three iso-caloric meals/d to maintain body weight. The two WL diet periods (HFWL and HPWL) were fed to 100 % RMR, to achieve caloric deficit, using a 7-d rotational menu (detailed in online Supplemental Tables 2–4). Participants consumed only three meals a day, with 45 % of daily calories consumed in the morning (big breakfast) and 20 % of calories consumed at the evening meal. To allow for some behavioural (appetite) adaptation, the lunch meal could be consumed *ad libitum* up to 35 % of daily calories as provided. The HFWL diet (35 % fat, 15 % protein and 50 % carbohydrate) contained at least 30 g/d total dietary fibre for a 2000 kcal intake and was provided as mixed soluble and insoluble fibre sources to maintain palatability of the diet (e.g. wheat bran, fava bean, lentil and buckwheat). The HPWL diet (35 % fat, 30 % protein and 35 % carbohydrate) contained a mixed meat matrix to include poultry, fish, red meat, eggs and dairy. Total dietary fibre was no more than 15 g/d for a 2000 kcal intake. The composition of each meal, in terms of energy, fat, carbohydrate and protein, was calculated by using an electronic version of McCance and Widdowson’s the composition of foods^(29)^; WinDiets software (Professional Version, Robert Gordon University, Aberdeen, UK, 2017). Nine participants consumed the HPWL diet first and ten participants consumed the HFWL first. The composition of the three study diets (MT, HPWL and HFWL) can be seen in Table 2. At the beginning of the study, participants received a four-day *ad libitum* food diary to record details of all habitual foods and beverages (g) using calibrated kitchen scales provided (Disc Electronic Kitchen Scale 1036, Salter Housewares). During dietary intervention, participants attended the Human Intervention Studies Unit three times a week, for a cooked breakfast meal and provision of all the prepared food items and drinks to re-heat at home. A weighed record was completed throughout the study for leftovers (g). Caffeine consumption was not allowed during the study, with decaf drinks provided.

**Table 2.** Macronutrient composition of the daily food consumed by overweight adults during the study MT, HFWL and HPWL dietary periods Table 2 long description.

Diet	Energy (kcal/d)	Fat (%^*^)	Protein (%^*^)	CHO (%^*^)	Alcohol (%^*^,^†^)	Fibre (%^*^)	Fibre (g)
Baseline	2170^b^	32·9^b^	17·1^b^	45·9^b^	2·09^b^	2·03^c^	22·5^b^
MT	2850^c^	30·2^a^	15·1^a^	52·7^c^	0·16^a^	1·86^b^	27·7^c^
HFWL	1880^a^	35·0^c^	15·1^a^	47·1^b^	0·03^a^	2·87^d^	28·2^c^
HPWL	1900^a^	34·9^c^	29·9^c^	33·9^a^	0·07^a^	1·32^a^	13·1^a^
SED	96	0·99	0·67	0·91	0·55	0·08	1·34
*P* ^‡^	< 0·001	< 0·001	< 0·001	< 0·001	< 0·001	< 0·001	< 0·001

Abbreviations: MT, Maintenance diet; HFWL, High-fibre weight loss diet; HPWL, High-protein weight loss diet; CHO, carbohydrate.

* Expressed as percentage of total energy.

† Alcohol from red wine used as an ingredient in one of the dinners on each diet.

‡ Analysed for diet effect by ANOVA, means in the same row not sharing a superscript are significantly different (*P* < 0·05). SED is based on within-participant spread.

### Test day measurements

Before all the tests, the participants fasted overnight (10 h). During each Type A, test day body density, waist and hip circumferences, RMR and the thermic effect of food (TEF) were measured. At each Type B test day, blood pressure, venous blood samples, GE, subjective appetite and total body water were measured. Measurements of body composition and metabolic rate were performed under standardised fasted conditions as described previously^(9)^. In brief, the three-compartment model of body composition^(30)^ was applied to the dataset, using measured total body water (by ^2^H dilution in plasma samples) and body density (Bod Pod Body Composition System, COSMED). In addition, body weight was measured three times a week during WL, with subjects wearing a previously weighed dressing gown.

### Resting and postprandial energy expenditure

RMR was measured fasting using the ventilated hood system. The TEF was assessed over a 4-h period after consumption of a test breakfast for 10 min every 30 min, as described previously^(9)^. A Microsoft Excel macro was used to calculate the postprandial energy expenditure (EE) at each time point by determining the consecutive data (minimum of 5 min) with the lowest CV within each 10 min measure. The postprandial increase in EE was analysed as AUC using the trapezoidal rule to calculate TEF^(31)^. Two QUARK systems were used on the study with the same machine being used for all participants’ tests unless technical or scheduling issues made this impossible.

The composition of the test breakfast meals on each Type A and Type B test day was the same as on other days (within each dietary period, Table 2), fed to 33 % of daily energy requirements for the MT diet and 45 % of daily calories for both WL diets (HFWL and HPWL). Food items provided on these sessions included cereal with milk, omelette, bacon, toast, baked beans, orange juice and fruit smoothies.

### Metabolic profile and glucose homeostasis

Blood pressure was monitored during screening and at the end of each dietary intervention period with the use of an automated system (Omron M5–1; Omron Healthcare Inc). After 30 min at rest in the semi-supine position, the average of three measures taken was recorded.

Before and after a test breakfast, blood samples were collected from a cannula in the arm, in Lithium Heparin tubes (BD Vacutainer^®^, Cat #367885, BD Diagnostics) and K_2_EDTA tubes (BD Vacutainer^®^, Cat #367873, BD Diagnostics). Samples were collected before the test meal (0 min) and postprandially at 180 and 360 min, a total of 33 mL full blood per test day from each participant. The samples were centrifuged at 1000 g for 15 min at 4℃. Before centrifugation, 160 µl of a previously prepared inhibitor mix containing (2-aminoethyl)-benzenesulfonyl fluoride hydrochloride (Roche Diagnostics, Basel, Switzerland), dipeptidyl peptidase IV soluble inhibitor (Merck Millipore, Watford, UK) and protease inhibitor cocktail (Sigma Aldrich) were added into the K+-EDTA sample tube. The plasma from the Lithium Heparin tubes was then aliquoted and stored at –70°C until analysis. Each participant’s samples were analysed in the same run, for the determination of glucose and lipid profile using KONELAB™ (Thermo Scientific) performed at the University of Aberdeen, as previously described^(32)^.

Plasma from the K_2_EDTA blood samples was used for the determination of fasted insulin using ELISA (Mercodia). This analysis was performed at the Rowett Institute. The immunoassay kit has a range of 3–200 mU/l (0·13–8·67 μg/l) with a detection limit of 1 mU/l. Furthermore, the mean recovery rate was 104 % and CV ranges from 2·6 % to 3·6 %. The fasted glucose & insulin results were applied to the homeostatic model assessment^(33)^ to calculate insulin resistance, b-cell function and the insulin:glucose ratio.

### Total body water analysis

Total body water was measured by ^2^H dilution. Participants were dosed before each test breakfast with > 0·12 g/kg body water of 99·8 % ^2^H. Plasma from the blood samples collected as described above (pre- and 6 h after breakfast) was analysed with MS for isotopic enrichment of the 6-h plasma samples relative to background enrichment at Maastricht University, Netherlands.

### Gut hormone analysis

The collection period of 6 h allowed capture of the *late* postprandial hormone exposure, for example, prolonged glucagon-like peptide-1 (GLP-1)/peptide YY (PYY) elevation after high-protein meal. Plasma from the K_2_EDTA samples was also analysed at the NIHR Core Biochemistry Assay Laboratory (Cambridge Biomedical Research Centre) for total ghrelin, glucose-dependent insulinotropic polypeptide (GIP), GLP-1 and PYY. Samples were measured by sandwich immunoassay using EMD Millipore Corporation, St Louis, USA (ghrelin) and MesoScale Discovery (MSD), Rockville, USA (GIP, GLP-1 and PYY) kits. This method has a lower limit of detection of 82, 5, 2 and 27 pg/ml for ghrelin, GIP, GLP-1 and PYY, respectively. Full details of the immunoassays are provided in the Supplementary Methods.

### Assessment of gastric emptying

Eighteen sets of breath samples were collected over 6 h postprandially, to assess GE using the ^13^C octanoic acid stable isotopic technique^(34)^. To collect five samples per timepoint, the participants blew through a plastic straw into evacuated Exetainer breath vials (Labco Limited) which were then sealed. Rate of CO_2_ production (*P*, mmol/min) per participant was estimated from body surface area using a height-weight formula (see Supplementary Methods for details). ^13^CO_2_ enrichments were expressed as atom percent excess and were corrected for baseline atom percent excess. The data were fitted to the mkβ model presented in Ghoos *et al.* ^(35)^ to acquire parameters for statistical analysis. Full details of the model are provided as a technical annex in Supplementary Methods.

### Assessment of appetite

Appetite was assessed on test days by use of pen and paper 100 mm visual analogue scales, applied before eating and every 30 min thereafter, as described previously^(36)^. In short, the questionnaires contained six questions related to motivation to eat: hunger, fullness, prospective consumption (quantity), desire to eat, thirst and preoccupation with thoughts of food. The scales were recording from, for example, ‘not at all hungry’ to ‘as hungry as I ever felt’ so that higher scores indicated more intense subjective sensations. A summary measure of subjective appetite was calculated from the responses to the questionnaires using the formula^(37)^:

$$\begin{array}{rl} Appetite\;score= & [hunger+(100-fullness)\\ & +prospective\;consumption+desire\;to\;eat]/4 \end{array}$$

The postprandial changes in appetite parameters were analysed as AUC using the trapezoidal rule.

### Faecal sample analysis

Faecal samples were analysed to determine gut microbiota composition, using quantitative real-time PCR, 16S rRNA gene amplicon sequencing and SCFA production. In addition to this, the number of bowel movements per day throughout the study and the appearance of the weekly stool samples, according to the Bristol Stool Form Scale^(38)^, were assessed by laboratory staff. Samples were collected by the participants into a pre-lined faecal collection pot. (Fecotainer^®^, AT Medical BV). They were required to return any samples to the institute within 16 h of production, for effective microbial microbiota and metabolome profiling. One participant was unable to provide a complete set of samples, so analysis presented is for *n* 18 only.

Faecal SCFA concentrations were determined as described previously^(39)^. After derivatisation, 1 µl of sample was analysed using a Hewlett-Packard gas chromatograph fitted with a silica capillary column with helium as a carrier gas. The SCFA concentrations were calculated from the relative response factor with respect to the internal standard 2-ethylbutyrate.

### Microbiome analyses

Full details, statistical methods and references for the faecal sample DNA extraction and subsequent quantitative real-time PCR and 16S rRNA gene amplicon sequencing are provided as a technical annex in the Supplementary Methods. The sequence data from this study are available in the European Nucleotide Archive under study accession number ERP121324, with samples registered as sample accessions ERS4531430-ERS4531602 (online Supplementary Table 5).

### Statistical analysis

Computer-generated random numbers were used to assign the participants to first receive either the HFWL or HPWL diet. Data on energy intake, EE, body weight and composition and blood metabolites were analysed by hierarchical ANOVA (linear mixed model for unbalanced data), with participant as blocking factors (random effect) and diet (MT, HFWL and HPWL) as treatment term (fixed effect). When the effect of diet was significant (*P* < 0·05), means were compared with post hoc *t* tests. Data on appetite ratings were analysed by mixed models, with random effect terms for participant, period within-participant, day within period and hour within day, and fixed effect terms for diet, day of intervention, time of day (by clock time) and their interactions.

Data on GE were assessed by AUC and CO_2_ excretion parameters, obtained from fitting models to the breath samples comprised of one value per diet per participant. These were analysed as one-way ANOVA with participant as random effect and diet as fixed effect, using contrasts (MT *v*. WL and HFWL *v*. HPWL) to test comparisons of interest. Normality was assessed by inspection of histograms of variables and residuals. If variables showed indications of skewness, analysis was repeated on a log scale to confirm that conclusions remained unchanged. Random effects regression was employed to investigate the effects of gut hormones on GE and appetite parameters, where each outcome was summarised by their AUC from 0 to 360 min.

All analyses were conducted using GenStat 17 Release 17·1 (Lawes Agricultural Trust, VSN International Ltd). All results are reported as Mean ± SED unless specified otherwise.

### Power analysis

Our primary objective was to examine differences in energy balance (body weight). Based on previous studies, within-group differences due to diet, for a WL of 1·4 kg and a SD of 1·4 kg^(4)^, twenty subjects were required to obtain 80 % power with a 5 % significance level. Twenty participants were also deemed adequate to assess changes in other key outcome measures (RMR, TEF and GE) based on 3 % variability in between-day RMR^(40)^ and 44 % reduction in TEF^(41)^ based on time of day eating and reported variability of GE using mass spectrometer measurements^(40)^. The primary outcome was change in energy balance, assessed by measuring body weight changes. The secondary outcomes were indices of gut health (assessed by changes in the composition of gut microbiota and concentrations of SCFA in faecal samples) and subjective appetite ratings (hunger, fullness, prospective consumption and desire to eat) assessed with visual analogue scales. Exploratory outcomes were anthropometric variables – BMI, RMR, fat mass, fat-free mass (FFM), waist circumference, hip circumference, waist–hip ratio, systolic and diastolic blood pressure, pulse; macronutrient intake during each dietary period; metabolic profile – fasting glucose, fasting insulin, total cholesterol, HDL-cholesterol, LDL-cholesterol, LDL:HDL ratio, TAG, total cholesterol: HDL ratio, NEFA; postprandial release of plasma glucose and gut hormones (ghrelin, GIP, GLP-1 and PYY) expressed over 360 min and as AUC; GE. All outcomes were assessed at the end of each dietary period.

## Results

### Participant characteristics

Nineteen subjects completed the study, and their baseline anthropometric characteristics are presented in Table 1.

### Impact of diet intervention on energy intake

The macronutrient composition of the study diets consumed can be seen in Table 2. There was no difference in energy intake for both WL diets, with no significant difference in the *ad libitum* lunch intake, with 651 kcal/d and 673 kcal/d consumed for lunch on the HFWL and HPWL diets, respectively (*P* = 0·225, SED 17 kcal/d).

### Impact of diet on body weight and body composition

**Figure 3.**
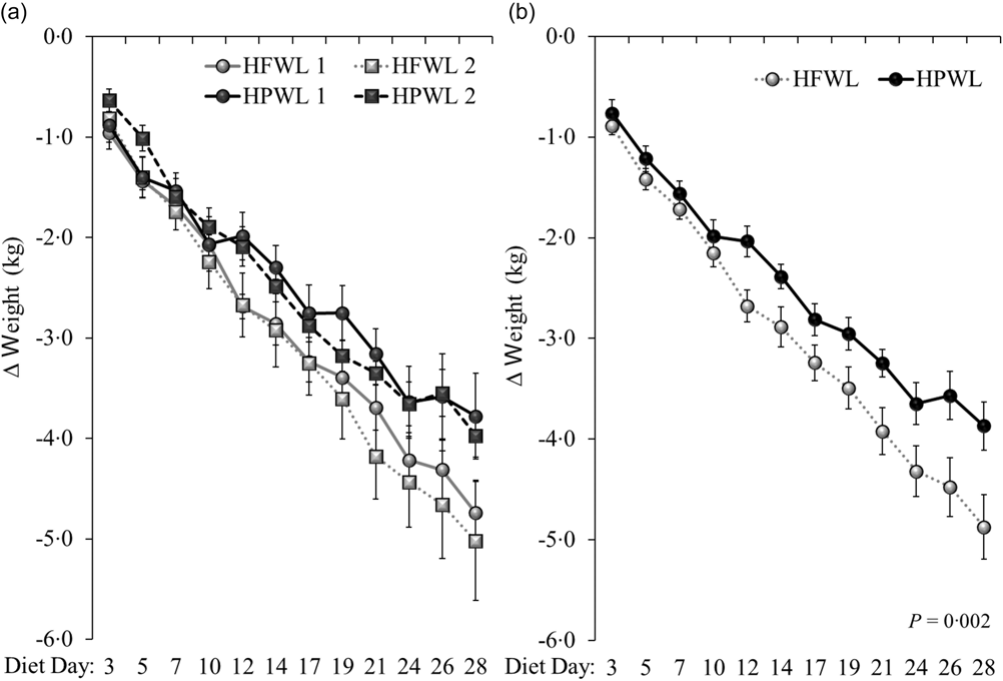
Comparison of body weight change after consumption of HFWL and HPWL diets. (a) By diet order and (b) by diet^1^. ^1^ *n* 19, data are expressed as means, and error bars are sem. Differences are considered significant if *P* < 0·05. Abbreviations: HFWL, High-fibre weight loss diet; HPWL, High-protein weight loss diet.

There was no significant effect of the order in which the two WL treatments occurred and no interaction with the diet effect (Figure 3(a)). There was a diet effect on WL (Figure 3(b)), with a mean WL of –4·87 kg and –3·87 kg for the HFWL and HPWL diets, respectively (*P* = 0·002, Table 3). Both diets also resulted in significant reductions in absolute and percentage fat mass (kg: HFWL –13·0 %, HPWL diet –14·6 %; %fat mass: HFWL –8·3 %, HPWL diet –9·9 %) relative to the MT diet (Table 4). FFM (kg) was also significantly reduced during both WL diets relative to the MT diet (HFWL –2·97 %, HPWL –1·74 %) with a greater reduction after the HFWL compared with the HPWL diet. There were no significant differences observed after consumption of the three diets for protein and mineral mass, or FFM hydration factor, derived from the three-compartment model. However, total body water volume was significantly lower after the HFWL diet (–3·22 %, −1·55 L) relative to the MT diet (*P* = 0·002, SED 0·40) with no significant difference in the HPWL diet relative to MT diet (−1·56 %, –0·75 L). This may have contributed to the difference in FFM loss between the diets. (Table 4). Significant reductions in waist and hip circumference and waist–hip ratio were also observed following both WL diets relative to MT diet with no differences between the HFWL and HPWL diets (Table 3).

**Table 3.** Mean anthropometric data of overweight adults after consumption of MT, HFWL and HPWL diets Table 3 long description.

Diet	MT	HFWL	HPWL	SED	*P* ^*^
Weight (kg)	102·2^b^	95·7^a^	96·1^a^	0·72	< 0·001
BMI (kg/m^2^)	33·2^b^	31·1^a^	31·2^a^	0·24	< 0·001
Weight loss (kg)	−0·65^c^	−4·87^a^	−3·87^b^	0·82	< 0·001
Body surface area (m^2^)	2·26^b^	2·18^a^	2·19^a^	0·01	< 0·001
SBP (mmHg)	136^b^	124^a^	123^a^	2·58	< 0·001
DBP (mmHg)	82·8^b^	76·3^a^	74·1^a^	1·39	< 0·001
Pulse (beats/min)	71·2^c^	64·1^ab^	63·1^a^	1·59	< 0·001
Waist circumference (cm)	112^b^	106^a^	106^a^	0·99	< 0·001
Hip circumference (cm)	111^b^	108^a^	107^a^	0·66	< 0·001
WHR	1·01^b^	0·98^a^	0·99^a^	0·01	0·002

Abbreviations: MT, Maintenance diet; HFWL, High-fibre weight loss diet; HPWL, High-protein weight loss diet; SBP, Systolic blood pressure; DBP, Diastolic blood pressure; WHR, Waist–hip ratio.

* Analysed for diet effect by ANOVA, means in the same row not sharing a superscript are significantly different (*P* < 0·05). SED is based on within-participant spread.

**Table 4.** Total body water and three-compartment model results of overweight adults after consumption of MT, HFWL and HPWL diets Table 4 long description.

Diet		MT	HFWL	HPWL	SED	*P* ^*^
H_2_O volume at 36°C (L)		48·1^b^	46·6^a^	47·4^ab^	0·40	0·002
FM	(kg)	36·6^b^	31·8^a^	31·2^a^	0·81	< 0·001
	(%)	34·9^b^	32·0^a^	31·5^a^	0·67	
FFM	(kg)	66·3^c^	64·3^a^	65·1^b^	0·39	< 0·001
	(%)	65·1^a^	68·0^b^	68·6^b^	0·67	
Protein mineral mass (kg)		18·4	18·0	18·0	0·21	0·081
FFM hydration factor		0·721	0·719	0·722	0·003	0·608

Abbreviations: MT, Maintenance diet; HFWL, High-fibre weight loss diet; HPWL, High-protein weight loss diet; FM, Fat mass; FFM, Fat-free mass.

* Analysed for diet effect by ANOVA, means in the same row not sharing a superscript are significantly different (*P* < 0·05). SED is based on within-participant spread.

### Impact of diet on appetite and motivation to eat

**Figure 4.**
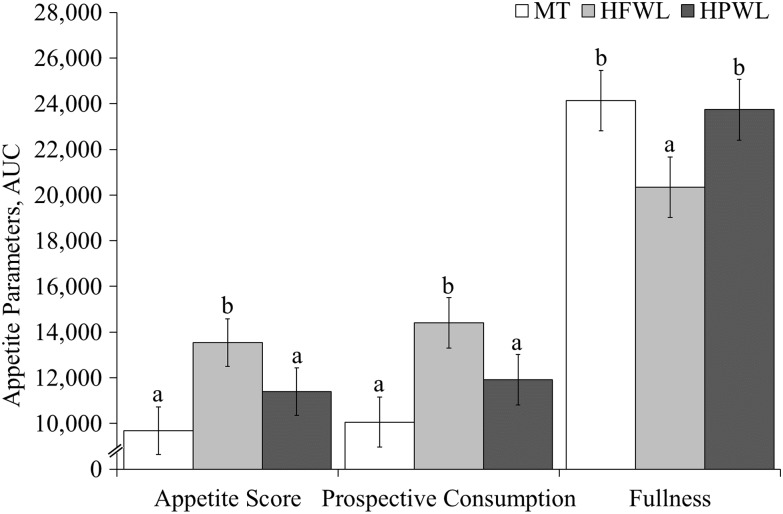
Comparison of AUC for appetite parameters of overweight adults after consumption of HFWL and HPWL diets^1^. ^1^ *n* 19, data are expressed as means, and error bars are SED. Means within a parameter not sharing a superscript are significantly different (*P* < 0·05). Abbreviations: HFWL, High-fibre weight loss diet; HPWL, High-protein weight loss diet.

The composition of the WL diet significantly impacted appetite visual analogue scale ratings across the test day. Relative to the HFWL meal, AUC after the HPWL meal was significantly lower for appetite score (–16 %, *P* = 0·003, SED 1040) and prospective consumption (–17 %, *P* = 0·001, SED 1100) and significantly higher for fullness (17 %, *P* = 0·013, SED 1320) (Figure 4). These results indicate that despite there being a reduced energy intake from both WL meals, the HPWL meal was better able to maintain the satiating capacity, while the HFWL meal resulted in lower postprandial satiety.

### Impact of diet on RMR and thermic effect of food

**Figure 5.**
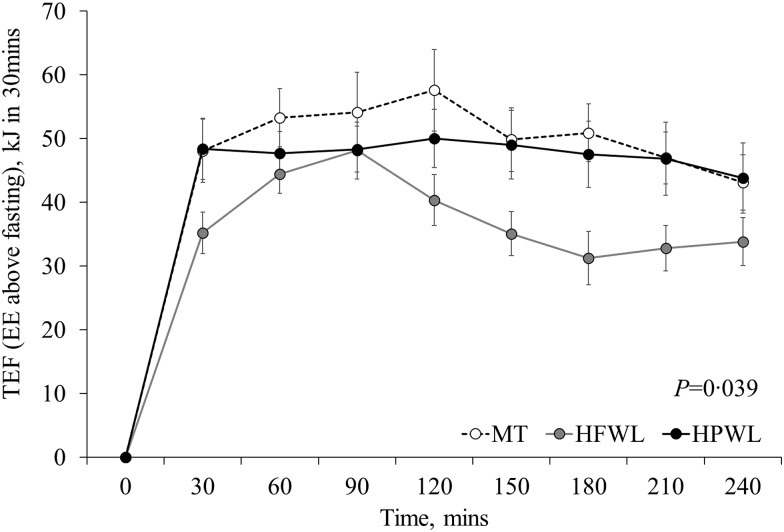
TEF after consumption of MT, HFWL and HPWL test meals^1^. ^1^ *n* 19, data are expressed as means, and error bars are sem. Differences are considered significant if *P* < 0·05. Abbreviations: EE, Energy expenditure; HFWL, High-fibre weight loss diet; HPWL, High-protein weight loss diet; MT, Maintenance diet; TEF, Thermic effect of food.

Associated with WL, there was a significant reduction in fasted RMR observed after both the HPWL (–8·12 %) and HFWL diets (–7·12 %) diets, relative to baseline (MT diet) (*P* < 0·001, SED 219, Table 5). There was no evidence of diet effect for the reduction in RMR observed for HPWL and HFWL diets. Interestingly, TEF (kJ) was significantly lower after the HFWL meal (–25 %) relative to both the MT and HPWL meals (*P* = 0·006, SED 29, Table 5. Also indicated in Figure 5 showing TEF results measured over 4 h *P* = 0·039). No difference was observed after the HPWL meal relative to the MT meal, even though it was a smaller energy load, likely reflecting the higher protein content.

**Table 5.** Comparison of RMR and TEF results after consumption of MT, HFWL and HPWL test meals Table 5 long description.

Test meal		MT	HFWL	HPWL	SED	*P* ^*^
Predicted RMR^†^	(kcal/d)	2020^b^	1890^a^	1910^a^	16	< 0·001
100 % RMR^‡^	(kJ/d)	8690^b^	8070^a^	7980^a^	219	< 0·001
	(kcal/d)	2080^b^	1930^a^	1900^a^	52	< 0·001
Meal Energy	(kJ)	4010^b^	3570^a^	3530^a^	103	< 0·001
Fat	(g)	32·2	33·3	33·7	1·00	0·29
	(kJ)	1190	1230	1250	37	0·29
	(% Energy)	29·9^a^	34·9^b^	35·4^c^	0·20	< 0·001
Protein	(g)	34·8^b^	31·1^a^	62·5^c^	1·66	< 0·001
	(kJ)	591^b^	529^a^	1060^c^	28	< 0·001
	(% Energy)	14·9^a^	15·0^a^	30·1^b^	0·39	< 0·001
CHO	(g)	134^c^	104^b^	73^a^	3·18	< 0·001
	(kJ)	2140^c^	1670^b^	1180^a^	51	< 0·001
	(% Energy)	53·8^c^	47·3^b^	33·2^a^	0·42	< 0·001
Fibre	(g)	6·91^b^	12·2^c^	6·10^a^	0·38	< 0·001
	(kJ)	55^b^	98^c^	49^a^	3	< 0·001
	(% Energy)	1·40^a^	2·78^b^	1·38^a^	0·03	< 0·001
Dinner (evening before test day)	(kJ)	3970^b^	1710^a^	1600^a^	140	< 0·001
TEF	(kJ)	381^b^	284^a^	355^b^	29	0·006

Abbreviations: TEF, Thermic effect of food; MT, Maintenance diet; HFWL, High-fibre weight loss diet; HPWL, High-protein weight loss diet; CHO, carbohydrate.

* Analysed for meal effect by ANOVA, means in the same row not sharing a superscript are significantly different (*P* < 0·05). SED is based on within-participant spread.

† Calculated with a regression equation from measured RMR, fat mass and fat-free mass.

‡ Measured by QUARK ventilated hood system.

### Influence of diet and test meal on cardiometabolic health blood indices

Overall, clear cardiometabolic improvements were evident, as a result of WL. WL significantly reduced systolic and diastolic blood pressure compared with the baseline (MT) diet, with no differences between the HFWL and HPWL diets (Table 3). The effects of the diets on fasting and postprandial blood lipids and glucose are reported in Table 6. Both WL diets created significant decreases in fasting and postprandial lipids; except for fasted HDL-cholesterol and NEFA, compared with baseline. There were no significant differences between the HFWL and HPWL diets. The only parameter that showed a significant difference by time × meal was TAG (Table 6). The blood values were higher at all three timepoints on the MT test day relative to the HPWL and HFWL test days (*P* < 0·001, SED 0·16). At 180 min, the HPWL TAG results were 26·5 % and 44·9 % lower than the same time point on the HFWL and MT test days, respectively (*P* < 0·001, SED 0·16). A diet effect was observed for fasted (Time 0) and postprandial plasma glucose concentrations, being 10·2 % and 8·4 % (Time 0, fasting) and 10·0 % and 6·9 % (average of three time points after eating) lower after the HFWL and HPWL diets, respectively, relative to after the MT diet. There were no significant differences observed between the HFWL and HPWL meals. Fasted insulin concentration (Time 0) was significantly lower after the HFWL and HPWL diets (28·4 % and 33·2 %, respectively) relative to after the MT diet (*P* < 0·001, SED 0·58). homeostatic model assessment of insulin resistance was significantly lower after the HFWL and HPWL diets (34·9 % and 38·4 %, respectively) relative to after the MT diet (*P* < 0·001, SED 5·92). Homeostatic model assessment of *β*-cell function was significantly lower after the HPWL diet relative to after the MT diet (20·8 %, *P* < 0·001, SED 0·58) but there was no significant difference after the HFWL diet relative to after the MT diet. Insulin:glucose ratio was significantly lower after the HFWL and HPWL diets (21·7 % and 28·6 %, respectively) relative to after the MT diet (*P* < 0·001, SED 0·10). There were no significant differences in insulin:glucose ratio observed between the HFWL diet relative to the HPWL diet. The variables showed slight indications of skewness, so the analysis was repeated on a log scale to confirm that the conclusions remained unchanged.

**Table 6.** Comparison of lipid and glucose results of overweight fasted adults (time 0) and after consumption of MT, HFWL and HPWL meals on the test day Table 6 long description.

Test meal	Time (min)	MT	HFWL	HPWL	SED_*meal*_	SED_*time.meal*_	*P*_meal_^*^	*P*_time.meal_^*^
Cholesterol (mmol/l)	0	5·48^b^	4·53^a^	4·61^a^	0·20	0·19	< 0·001	0·474
	180	5·36	4·51	4·54				
	360	5·66	4·53	4·74				
	ALL	5·50^b^	4·52^a^	4·63^a^	0·17		< 0·001	
HDL-cholesterol (mmol/l)	0	1·12	1·14	1·17	0·05	0·04	0·571	0·885
	180	1·10	1·11	1·13				
	360	1·10	1·09	1·11				
	ALL	1·11	1·11	1·14	0·04		0·726	
LDL-cholesterol (mmol/l)	0	3·38^b^	2·58^a^	2·71^a^	0·15	0·16	< 0·001	0·697
	180	3·14	2·49	2·62				
	360	3·34	2·56	2·70				
	ALL	3·29^b^	2·54^a^	2·67^a^	0·14		< 0·001	
TC-HDL-cholesterol ratio	0	5·06^b^	4·35^a^	4·16^a^	0·26	0·28	0·003	0·779
	180	5·12	4·44	4·23				
	360	5·43	4·60	4·58				
	ALL	5·20^b^	4·47^a^	4·32^a^	0·26		0·004	
LDL-HDL-cholesterol ratio	0	3·11^b^	2·49^a^	2·46^a^	0·18	0·20	0·001	0·894
	180	3·00	2·46	2·45				
	360	3·18	2·60	2·60				
	ALL	3·09^b^	2·52^a^	2·50^a^	0·19		0·004	
NEFA (mmol/l)	0	0·61	0·72	0·72	0·06	0·05	0·115	0·297
	180	0·21	0·24	0·23				
	360	0·57	0·64	0·54				
	ALL	0·46	0·54	0·50	0·03		0·082	
TAG (mmol/l)	0	2·07^b^	1·53^a^	1·27^a^	0·13	0·16	< 0·001	< 0·001
	180	2·67	2·00	1·47				
	360	2·51	1·88	1·80				
	ALL	2·42^b^	1·80^a^	1·52^a^	0·15		< 0·001	
Glucose (mmol/l)	0	5·97^b^	5·36^a^	5·47^a^	0·14	0·26	< 0·001	0·091
	180	5·86	4·93	5·11				
	360	5·18	5·01	5·27				
	ALL	5·67^b^	5·10^a^	5·28^a^	0·18		0·011	
Insulin (mU/l)	0	9·52^b^	6·82^a^	6·36^a^	0·58		< 0·001	
HOMA-IR	0	2·55^b^	1·66^a^	1·57^a^	0·16		< 0·001	
HOMA-*β*cell	0	82·7^b^	76·0^ab^	65·5^a^	5·92		0·023	
IGR	0	1·61^b^	1·26^a^	1·15^a^	0·10		< 0·001	

Abbreviations: MT, Maintenance diet; HFWL, High-fibre weight loss diet; HPWL, High-protein weight loss diet; TC, Total cholesterol; HOMA-*β*cell, Homeostatic model assessment of *β*-cell function; HOMA-IR, Homeostatic model assessment of insulin resistance; IGR, Insulin:glucose ratio.

* Analysed by ANOVA, means in the same row not sharing a post hoc *t* test superscript are significantly different (*P* < 0·05). SED is based on within-participant spread.

### Influence of diet on gut hormone plasma profile

**Figure 6.**
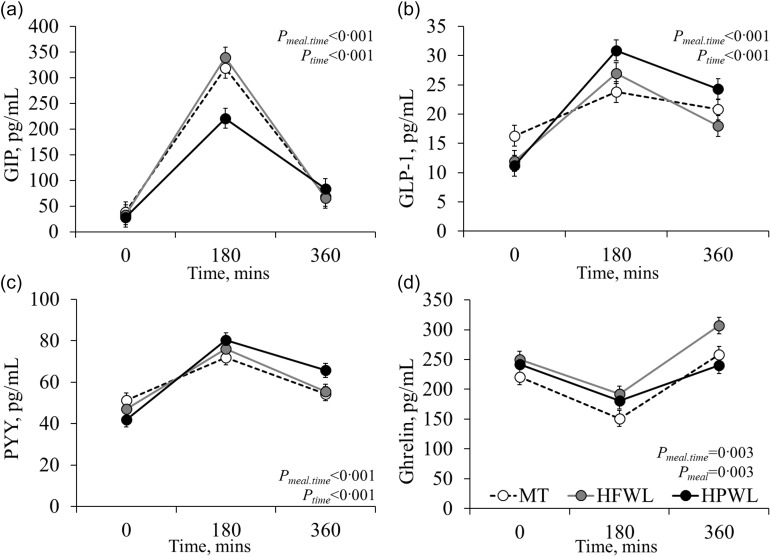
Figure 6 long description. Plasma gut hormone concentrations; (a) GIP, (b) GLP-1, (c) PYY and (d) ghrelin for overweight adults before and after consumption of MT, HFWL and HPWL test meals^1^. ^1^Data are expressed as means, *n* 19, and SED is based on within-participant spread. Differences and interactions are considered significant if *P* < 0·05. Abbreviations: GIP, Glucose-dependent insulinotropic polypeptide; GLP-1, Glucagon-like peptide-1; HFWL, High-fibre weight loss diet; HPWL, High-protein weight loss diet; MT, Maintenance diet; PYY, peptide YY.

**Figure 7.**
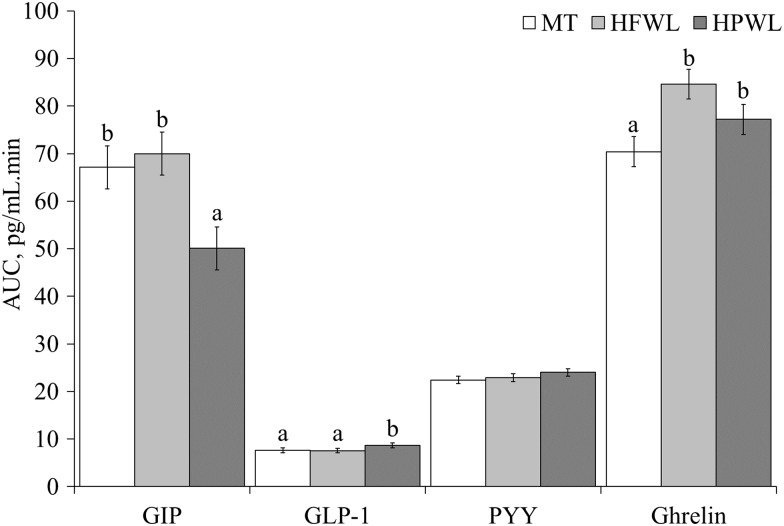
Comparison of AUC for plasma gut hormones; (a) GIP, (b) GLP-1, (c) PYY and (d) ghrelin for overweight adults after consumption of MT, HFWL and HPWL test meals^1^. ^1^ *n* 19, data are expressed as means, and error bars are SED. Means within a parameter not sharing a superscript are significantly different (*P* < 0·05). Abbreviations: GIP, Glucose-dependent insulinotropic polypeptide; GLP-1, Glucagon-like peptide-1; HFWL, High-fibre weight loss diet; HPWL, High-protein weight loss diet; MT, Maintenance diet; PYY, Peptide YY.

Gut hormones (GIP, GLP-1, PYY and ghrelin) were analysed in plasma over time per test meal, and the results can be seen in Figure 6 (absolute concentrations) and Figure 7 (AUC). The variables showed slight indications of skewness, so the analysis was repeated on a log scale to confirm that the conclusions remained unchanged.

GIP AUC was significantly lower on the HPWL diet compared with the MT (25·3 % lower) and HFWL (28·4 % lower) diets (*P* < 0·001, SED 4·52). In contrast, the HPWL diet had a significantly higher GLP-1 AUC (13·7 % and 14·8 % higher than MT and HFWL meal, respectively). The lower GIP AUC for the HPWL diet was primally the result of lower GIP at 180 min following consumption of the HPWL meal (30·6 % and 34·8 % relative to the MT and HFWL meals, respectively, *P* < 0·001, SED 19·5). Fasting GLP-1 concentrations were higher in the MT diet by 26·3 % and 31·0 % relative to the HFWL and HPWL meals, respectively (*P* < 0·001, SED 1·79). However, postprandial GLP-1 was increased more by the HPWL meal with concentrations at 180 min, 29·9 % and 14·8 % higher relative to after the MT and HFWL meals, respectively (*P* < 0·001, SED 1·79).

PYY at 360 min was also significantly elevated after the HPWL meal by 20·7 % and 18·2 % relative to after the MT and HFWL meal, respectively (*P* < 0·001, SED 3·44), yet did not lend itself to significant differences in PYY AUC. Ghrelin AUC was significantly higher for both WL diets compared with MT diet (*P* = 0·003, SED 3·16). At 360 min postprandial, the HFWL diet had significantly higher ghrelin levels by 19·0 % and 27·9 % relative to after the MT and HPWL meals, respectively (*P* = 0·003, SED 13·6). This likely contributed to the significantly higher test day average by 18·7 % and 11·4 % relative to after the MT and HPWL meals, respectively (*P* = 0·003, SED 9·24). There were no differences between diets in the test day average for GLP-1 or PYY (data not shown).

As expected, the effect of time was significant for all parameters with the highest values of GIP (*P* < 0·001, SED 12·3), GLP-1 (*P* < 0·001, SED 0·88), PYY (*P* < 0·001, SED 1·97) and the lowest values of ghrelin (*P* < 0·001, SED 7·09) recorded 180 min post-meal.

### Influence of diet on gastric emptying

GE was significantly affected by WL and diet composition (Table 7). There was a significant effect of WL on *t*_*0·5*_, with an extended duration until half emptying time was reached in both the HFWL and HPWL diets relative to MT diet (*P* = 0·013). Considering the modestly reduced meal energy content of the WL diets (Table 5), this further emphasises the impact WL had on slowing GE. Despite a modestly longer *t*_*0·5*_ in the HP relative to the HF diet, this difference was not significant. The WL diets resulted in a significantly longer duration of rapid excretion *(t*_*asc*_), which was likely the result of a slightly early (NS) initial excretion time (*t*_*lat*_) in the HPWL diet and a later half-time. The HPWL meal led to a further extended *t*_*asc*_ duration compared with the HFWL diet (*P* = 0·030). There were no significant differences in time to reach maximum excretion rate (*t*_*lag*_) between the diets.

**Table 7.** Mean values of estimated accumulated ^13^CO_2_ model parameters of overweight adults after consumption of MT, HFWL and HPWL diets^*^ Table 7 long description.

	Diet	*t*_*lag*_ (min)	*t*_*0·5*_ (min)	*t*_*lat*_ (min)	*t*_*asc*_ (min)
Mean	MT	259	321	143	178
	HFWL	287	371	145	226
	HPWL	295	412	126	286
SED		22·2	31·2	12·4	26·4
*P*_diet_^†^		0·246	0·021	0·268	0·001
*P*_contrasts_^‡^	MT *v*. WL	0·103	0·013	0·536	0·002
	HPWL *v*. HFWL	0·735	0·201	0·135	0·030

Abbreviations: t_lag_, timepoint at which ^13^CO_2_ excretion rate in the breath is at its maximum; t_0·5_, half-time, timepoint at which 50 % of the total excretion of ^13^CO_2_ in the breath has been recovered; t_lat_, initial delay or latency of ^13^CO_2_ excretion in the breath; t_asc_, the length of time during which ^13^CO_2_ excretion in the breath is rapid, i.e., when the cumulative curve is ascending; MT, Maintenance diet; HFWL, High-fibre weight loss diet; HPWL, High-protein weight loss diet.

* *n* 19. Analysis was repeated on a log scale as distributions appeared skewed. Statistical results are from the log analysis.

† Analysed by ANOVA, means in the same row not sharing a superscript are significantly different (*P* < 0·05). SED is based on within-participant spread.

‡ From contrasts comparing MT *v*. the remaining two diets (MT *v*. WL) and comparing the HPWL diet against the HFWL diet (HPWL *v*. HFWL).

### Relationship between appetite measurements, gut hormones and gastric emptying

Ghrelin significantly correlated with all appetite parameters; hunger (0·08, *P* = 0·009), fullness (–0·22, *P* = 0·002), desire to eat (0·27, *P* < 0·001), prospective consumption (0·14, *P* = 0·003), preoccupation with food (0·12, *P* = 0·006) and appetite score (0·20, *P* < 0·001). GLP-1 significantly correlated with prospective consumption (–0·08, *P* = 0·026). No significant correlations were observed between GIP and appetite parameters or GE model parameters.

### Effect of diet on stool sample consistency and frequency

**Figure 8.**
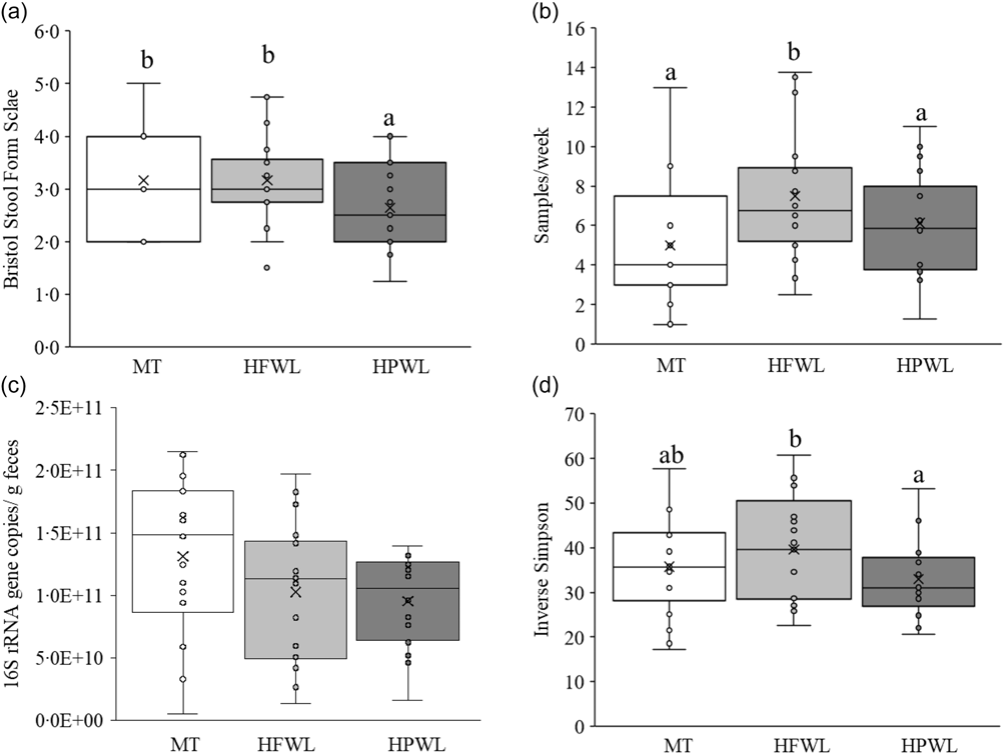
Figure 8 long description. Impact of diet on Bristol Stool Form Scale, stool frequency (samples/week), total faecal bacterial load (assessed using 16S rRNA gene copy numbers) and faecal microbiota diversity (using the inverse Simpson calculator)^1^. ^1^Presented for *n* 18 subjects, as one participant was unable to provide a complete set of faecal samples. Data are expressed as means, and error bars are SED. Means within a parameter not sharing a superscript are significantly different (*P* < 0·05). Abbreviations: HFWL, High-fibre weight loss diet; HPWL, High-protein weight loss diet; MT, Maintenance diet.

There were some statistically significant impacts on stool samples between different dietary intervention periods. Bristol Stool Form Scores were lower on the HPWL diet compared with the HFWL and MT dietary period (no significant difference between these latter two) (Figure 8(a)), while the number of stool samples passed per week was statistically higher on the HFWL diet compared with both the MT and HPWL dietary periods (Figure 8(b)).

### Dietary effect on faecal microbiota composition

Samples from the 7-d washout period between the HPWL and HFWL dietary interventions were initially compared with pre-intervention MT samples. These comparisons, using the Parsimony and AMOVA tests as implemented in the mothur software package^(42)^ as well as Satterthwaite’s method^(43)^, all showed no significant differences between washout and MT samples. The washout samples were therefore not included in further statistical analyses (final number of included samples = 156). The faecal microbiota profiles for the MT, HPWL and HFWL dietary periods were then compared against each other to see how total bacterial loads and the richness and evenness of bacterial species (*α* diversity) were affected by the changing diets and also how the composition of these species (*β* diversity) was affected. There was no significant difference in total bacterial loads between diets (*P* = 0·08, Figure 8(c)). The faecal microbiota *α* diversity on the HPWL diet was less diverse than when on the HFWL diet, as assessed by the Shannon and inverse Simpson diversity indices (*P* = 0·023 and 0·023 respectively, Figure 8(d)).

Faecal microbiota profiles were next compared with assess whether changing diets led to significant compositional changes under different diet regimes (*β* diversity). As expected, intra-individual variation was the strongest factor in determining the overall bacterial community compositions. Jaccard-based dendrogram (online Supplementary Figure 1) and Bray–Curtis-based principal coordinates analysis (online Supplementary Figure 2) both revealed that samples clustered most closely to others from the same participant, rather than by diet. However, both parsimony (*P* = < 0·001 with both Bray–Curtis and Jaccard calculators) and AMOVA (*P* = < 0·001 with Bray–Curtis) tests indicated that there were statistically significant differences in microbiota composition between HPWL and HFWL diets. The HPWL diet was also significantly different from the MT period using the Bray–Curtis AMOVA measure only (*P* = 0·014). In order to better understand the specific microbial taxa differences that underpinned these results, we next used Metastats and LEfSe to identify the most significant operational taxonomic unit and genus-level changes.

**Figure 9.**
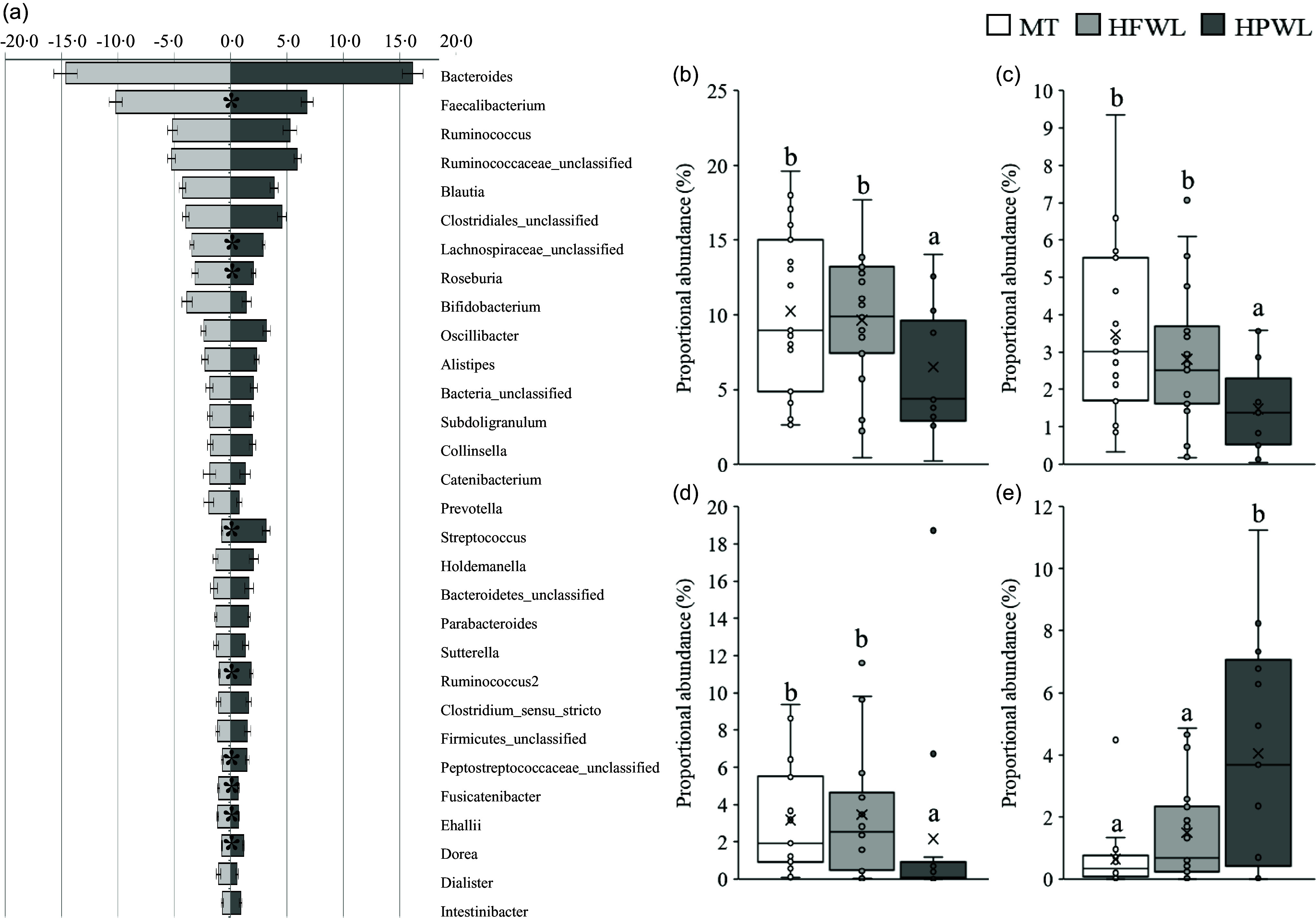
Compositional changes in faecal microbiota that were associated with changing diet. (a) Mean proportional abundance (% of total faecal microbiota sequences) of the top 30 genera when participants were consuming the HPWL and HFWL diets. Genera that were significantly different between dietary periods (as indicated by both LEfSe and Benjamini–Hochberg-corrected Metastats; *P* < 0·05) are shown with asterisks. (b)–(e) Box plots of the most statistically significantly different genera between the MT, HFWL and HPWL diets; (b) *Faecalibacterium*, (c) *Roseburia*, (d) *Bifidobacterium* and (e) *Streptococcus*. ^1^One participant was unable to provide a complete set of faecal samples, *n* 18. Abbreviations: HFWL, High-fibre weight loss diet; HPWL, High-protein weight loss diet; MT, Maintenance diet.

At the operational taxonomic unit level, the butyrate-producing bacteria *Faecalibacterium prausnitzii*, *Anaerostipes hadrus* and *Roseburia faecis* were all identified by Metastats and LEfSe (*P* < 0·05) as being associated with the HFWL diet. In contrast, the HPWL diet resulted in higher proportional abundances of *Streptococcus* spp. and *Escherichia coli*, as assessed by both Metastats and LEfSe approaches (data not shown). At the genus level, *Faecalibacterium*, *Roseburia* and *Bifidobacterium* were all significantly associated with the HFWL diet and *Streptococcus* with the HPWL diet (all significant with both Metastats and LEfSe). There were no significant differences in the proportional abundance of these four genera between the HFWL and MT dietary periods, but all four were significantly different between HPWL and MT. These results are summarised in Figure 9, with the top thirty most abundant bacterial genera presented alongside significant associations with diet.

### Effect of diet on faecal metabolites

**Figure 10.**
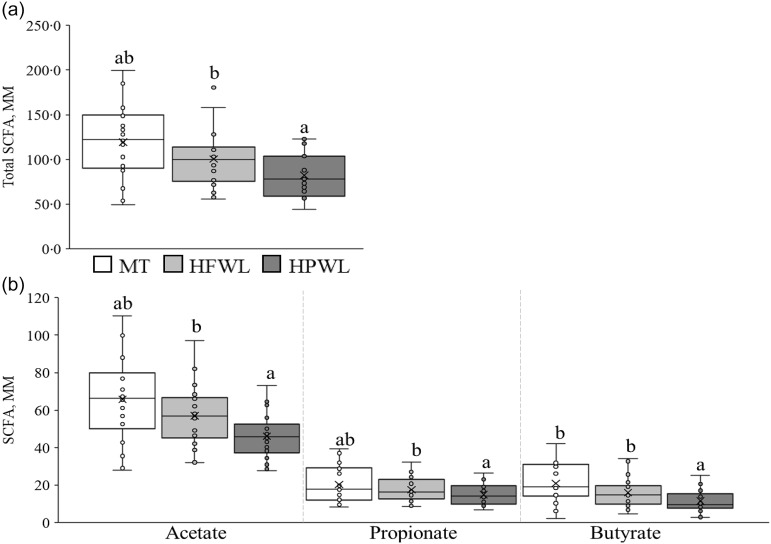
(a) Total SCFA and (b) individual SCFA concentrations in stool samples of study participants after consumption of MT, HFWL and HPWL diets^1^. ^1^Presented for *n* 18 subjects, as one participant was unable to provide a complete set of faecal samples. Data are expressed as means, and error bars are SED. Means within a parameter not sharing a superscript are significantly different (*P* < 0·05). Abbreviations: HFWL, High-fibre weight loss diet; HPWL, High-protein weight loss diet; MT, Maintenance diet.

Absolute concentrations of the major faecal SCFA acetate, propionate and butyrate, as well as total SCFA, were significantly lower on the HPWL compared with the HFWL diet (Figure 10), which is in agreement with the reduced fibre intake and lower relative abundance of key SCFA producers after HPWL intake. Due to the larger inter-individual variation of MT samples, only butyrate was significantly lower after HPWL intake compared with MT. In relative terms, there was a significant reduction in butyrate after HPWL compared with both MT and HFWL and a significant increase in branched-chain fatty acids and valerate (this did not reach significance compared with MT for valerate, online Supplementary Figure S3)

## Discussion

This work supports that the composition of breakfast meals is important as a means to improve WL and metabolic health, to meet specific health outcomes. While both HFWL and HPWL diets were both able to produce clinically significant WL and specifically fat mass loss, the diets differentially modified appetite and gut microbiota profile. The HPWL diet resulted in greater satiation which can be helpful under *ad libitum* conditions and for long-term dietary compliance. In contrast, the HFWL diet resulted in a superior gut microbiota profile, which may benefit gut health in the longer-term.

### Appetite control and diet composition

Both HPWL and HFWL diet have been promoted for their capacity to increase satiation^(26)^. One of the key findings from our study was the superior maintenance of satiety after the HP meal compared with the HFWL meals. HP diets fed *ad libitum* are effective at inducing WL and preventing weight regain^(17,44–46)^; however, effects on appetite during energy restriction are less consistent. Some studies have reported a superior capacity for protein intake to suppress appetite^(47–50)^, but a number report no differences between high- and low-protein groups^(51–53)^. Factors such as the timing of the protein intake, as well as the type of protein, may all differentially influence appetite control. Leidy *et al.* ^(21)^ suggested that during energy restriction, HP at breakfast led to greater postprandial satiation and whole-day satiety compared with HP provided at lunch or dinner time. A primary mechanism contributing to protein satiation is the satiety-stimulating effects of gastrointestinal peptides, in particular, GLP-1 and PYY^(54,55)^. Although we observed a significantly higher GLP-1 at 180 min after the HPWL diet compared with the HFWL diet and MT diet, there were no differences in PYY after any of the diets, and there was only a weak relationship between GLP-1 and prospective consumption, with no relationship to other subjective ratings of appetite. Interestingly, WL, regardless of diet, appeared to influence meal-induced GLP-1 release, whereby, despite a lower fasting GLP-1, the meal induced a notably larger increase in GLP-1 at 180 min. The increase in ghrelin is in line with other studies where, during energy restriction, increased gastric ghrelin and reduced postprandial appetite hormones can contribute to increased appetite^(44,56,57)^. The mechanisms by which fibre induces satiety include its capacity to increase viscosity and swelling in the gut, thereby increasing the activation of mechanoreceptors in response to gastric stretch and slowing GE. These physiological effects contribute significantly to the feeling of fullness after a meal^(58)^. In addition, undigested fibre can be fermented by the colonic microbiota, which has been proposed to reduce appetite through the release of SCFA and the subsequent activation of gut hormones^(59,60)^. The actions of fibres depend on their structure, where soluble fibres increase the viscosity of the contents of the upper gastrointestinal tract^(61,62)^, thereby reducing GE and inducing fullness through swelling in the gut and activation of mechanoreceptors in response to gastric stretch. We used a mix of soluble and insoluble fibres to ensure palatability of meals. Interestingly, previous studies assessing the effects of fibre on satiety are not always consistent^(63)^. Various factors influence the rate of GE, including metabolic, hormonal and neuronal signals. GLP-1 has been shown to elicit a potent ileal brake, largely slowing the rate of GE, primarily through activation of the vagus nerve^(64)^. GE half-time has been reported to be inversely related to GLP-1 following a carbohydrate meal. Cholecystokinin and PYY are also associated with GE, slowing GE via relaxing the proximal stomach and inhibiting antral motor activity. The effects of GE on appetite remain controversial, with some studies indicating that a faster GE is related to greater appetite suppression and others indicating slower^(65)^. We report no correlation between any of the GE parameters and subjective ratings of appetite indicating other factors may be at play.

### Diet composition and body composition

Previous studies support the notion that high-protein diets are superior for weight and fat mass loss^(66,67)^ while maintaining FFM^(68–70)^. While we report a small reduction in FFM at the end of both the HFWL and HPWL dietary periods, the slightly higher FFM after the HPWL diet appeared to have come from differences in total body water, with no differences in protein mineral mass.

### Diet composition and energy metabolism

As anticipated, RMR was significantly lower following both WL diets compared with baseline^(71)^. While reduced RMR is a typical response to WL, there were no differences between the HFWL or HPWL diets. The effects of the HPWL diet on EE were more notable in measurement of postprandial TEF. The HPWL meal resulted in a greater TEF compared with the HFWL meal. The HPWL meal was not significantly different from the MT meal despite being reduced energy intake. This higher TEF from protein is in agreement with the well-established evidence that protein results in a greater postprandial increase in EE^(17)^ likely as a result from increased postprandial protein synthesis and protein oxidation^(71)^. TEF has been reported to be higher in the biological morning^(41,72)^ and has been theorised to potentially contribute to the greater WL reported in previous studies where energy intake is more heavily distributed towards the morning^(4)^. We aimed to determine whether the merger of heightened morning TEF with HP TEF could enhance EE and differentially effect WL compared with HF breakfasts. While significant, there was only a 76 kJ difference between the TEF after the HFWL *v*. the HPWL diet^(73)^.

### Changes in faecal microbiota and metabolites

The microbiota analyses clearly demonstrated that both WL diets impacted on gut bacteria, which has relevance for wider gut health. The HPWL diet resulted in reduced microbiota diversity when compared with the HFWL diet, as shown by Shannon and inverse Simpson diversity indices, and there was also a reduction in the proportional abundance of a number of bacterial taxa with postulated health benefits. This includes a reduction in bifidobacteria, as well as key butyrate-producing groups such as *Faecalibacterium*, *Roseburia* and *Anaerostipes*. These findings agree with previous work that has also demonstrated that high-protein/low-fibre diets result in marked reduction of these bacteria and their beneficial metabolic products^(13,74)^. Butyrate has long been postulated to have important roles in maintenance of gut health, acting as an energy source for colonocytes, bolstering the gut barrier, and with potential anti-inflammatory and anti-carcinogenic properties^(75)^. Reduced overall bacterial diversity, as well as reduction of bifidobacteria and butyrate producers, has also been associated with several disease states^(76)^, indicating that there may be potential longer-term detrimental effects of the HP *v*. the HF approach to increasing satiety and WL. The HPWL diet was also associated with increased proportional abundance of *Streptococcus* and *Escherichia* species. Both genera contain opportunistic pathogens, but the 16S rRNA gene sequencing approach lacks the precision to determine whether these increases are likely to have had any ill effects on the host. Indeed, it is possible that the increased proportional abundance of the *Streptococcus* spp. (0·65 % and 0·77 % during the MT and HF dietary periods, respectively, *v*. 3·13 % on the HP diet) may simply reflect increased dietary consumption of these bacteria. *Streptococcus thermophilus* is a common starter culture used in the production of many dairy products, and the participant food diaries showed that during the HP diet, there was a higher consumption of yoghurt than the HF diet (around 50 g). The HF and HP diets also resulted in largely expected impacts on stool consistency and frequency, with the HF diet increasing the number of bowel movements, and higher Bristol Stool Form Scores, compared with HP. Reduced faecal SCFA concentrations after the HP diet were also in accordance with the lower-fibre intake on this diet and the concomitant reduction in relative abundance of key SCFA producers, as previously observed on lower-fibre diets^(13,74)^. Branched-chain fatty acids are generated from amino acids by gut bacteria and have been found before to increase on high-protein diets^(74,77)^.

### Study strengths and limitations

One limitation of this study is that it was conducted under free-living conditions, and this can increase the chances of participant noncompliance. However, given the regularity of attendance at the nutrition unit, provision of all meals and regular body weight checks, we believe any noncompliance not reported in food records was minimal and did not largely influence the study outcomes. It would also have been interesting to apply more advanced techniques to assess body composition. A further limitation was four weeks per diet; a longer period may yield different insights. Applying a within-subject cross-over design was also necessary to assess subjective changes in appetite and faecal gut health indices as a longer diet intervention would result in a higher participant burden. We introduced the washout to create a period of weight stability to collect baseline data in a similar state of energy balance, but one week may not be sufficient. For example, the reduction in RMR associated with WL would carry over to the second period to be cumulative, as subjects did not regain weight in this period. We have previously reported (Walker *et al.*, 2010)^(11)^ changes in gut microbiome composition in response to diet shifts, within days, being rapid and stable. The study design was applied as four weeks per diet treatment as being long enough to observe physiologically meaningful weight change but short enough to minimise dropout and keep dietary control feasible. There is scope for future studies to assess acute effects of chrono-nutrition on appetite control as within-day interventions, alongside longer-term parallel design studies, to explore the impact of diet composition/timing for sustained WL

Overall, our trial had many important strengths. It used many gold standard techniques to effectively measure appetite, energy balance and gut health.

### Conclusion

These data confirm that the composition of breakfast meals has an important role in influencing subjective appetite control, with the HP diet promoting greater feelings of satiety. Furthermore, the HF diet promoted a greater WL and proportional abundance of putatively beneficial groups of gut microbiota compared with HP diet, indicating that the former diet may be preferable for maintenance of gut health.

## Supporting information

Fyfe et al. supplementary material 1 Fyfe et al. supplementary material

Fyfe et al. supplementary material 2 Fyfe et al. supplementary material

Fyfe et al. supplementary material 3 Fyfe et al. supplementary material

Fyfe et al. supplementary material 4 Fyfe et al. supplementary material

Fyfe et al. supplementary material 5 Fyfe et al. supplementary material

Fyfe et al. supplementary material 6 Fyfe et al. supplementary material

Fyfe et al. supplementary material 7 Fyfe et al. supplementary material

Fyfe et al. supplementary material 8 Fyfe et al. supplementary material

Fyfe et al. supplementary material 9 Fyfe et al. supplementary material

## Supplementary material

For supplementary material/s referred to in this article, please visit https://doi.org/10.1017/S000711452610645X

## Figure 1 Long description

A flowchart illustrating the stages of participant flow in a study. The process begins with enrollment, where 198 participants are assessed for eligibility. 173 participants are excluded for not meeting inclusion criteria, declining to participate, or other reasons. 25 participants are randomly assigned to the intervention. In the allocation stage, 22 males and 3 females are allocated to the intervention. 19 males and 3 females receive the allocated intervention, while 3 males do not receive the intervention. During follow-up, 2 males and 1 female discontinue the intervention. In the analysis stage, 17 males and 2 females are analyzed, with 1 female excluded from the analysis due to incomplete faecal samples. The study is completed by 19 participants.

Navigate back to Figure 1.

## Figure 6 Long description

The figure contains four line graphs labeled (a), (b), (c), and (d), each depicting plasma gut hormone concentrations for overweight adults before and after consuming different test meals. Panel A: The line graph shows the concentration of GIP (Glucose-dependent insulinotropic polypeptide) in pg/mL over time in minutes. The x-axis represents time in minutes (0, 180, 360), and the y-axis represents GIP concentration in pg/mL. Three different test meals are represented: MT (Maintenance diet), HFWL (High-fibre weight loss diet), and HPWL (High-protein weight loss diet). The graph shows a peak in GIP concentration at 180 minutes, with significant differences based on meal time and overall time. Panel B: The line graph shows the concentration of GLP-1 (Glucagon-like peptide-1) in pg/mL over time in minutes. The x-axis represents time in minutes (0, 180, 360), and the y-axis represents GLP-1 concentration in pg/mL. Similar to Panel A, three different test meals are represented. The graph shows a peak in GLP-1 concentration at 180 minutes, with significant differences based on meal time and overall time. Panel C: The line graph shows the concentration of PYY (peptide YY) in pg/mL over time in minutes. The x-axis represents time in minutes (0, 180, 360), and the y-axis represents PYY concentration in pg/mL. The graph shows a peak in PYY concentration at 180 minutes, with significant differences based on meal time and overall time. Panel D: The line graph shows the concentration of ghrelin in pg/mL over time in minutes. The x-axis represents time in minutes (0, 180, 360), and the y-axis represents ghrelin concentration in pg/mL. The graph shows a decrease in ghrelin concentration at 180 minutes, with significant differences based on meal time and overall time.

Navigate back to Figure 6.

## Figure 8 Long description

The figure contains four box plots labeled (a) through (d), each representing different dietary impacts. Panel A shows a box plot of the Bristol Stool Form Scale with the y-axis labeled from 0 to 6. The x-axis categories are MT, HFWL, and HPWL. Panel B displays a box plot of stool frequency in samples per week, with the y-axis ranging from 0 to 16. The x-axis categories are MT, HFWL, and HPWL. Panel C presents a box plot of total faecal bacterial load measured in 16S rRNA gene copies per gram of feces, with the y-axis ranging from 0 to 2.5E+11. The x-axis categories are MT, HFWL, and HPWL. Panel D shows a box plot of faecal microbiota diversity using the inverse Simpson calculator, with the y-axis ranging from 0 to 70. The x-axis categories are MT, HFWL, and HPWL. Each box plot includes means and error bars representing SED, with significant differences indicated by different superscripts.

Navigate back to Figure 8.

## Table 1 Long description

A table summarizing baseline anthropometric characteristics of nineteen overweight and obese adults. The table has 18 rows and 5 columns. The columns are labeled Mean, SEM, Min, and Max. The rows are labeled with various characteristics such as Age (years), Height (m), Weight (kg), BMI (kg/m^2), SBP (mmHg), DBP (mmHg), Measured RMR (kJ/d), Measured RMR (kcal/d), Waist circumference (cm), Hip circumference (cm), WHR, Cholesterol (mmol/l), Glucose (mmol/l), HDL-cholesterol (mmol/l), LDL-cholesterol (mmol/l), NEFA (mmol/l), TAG (mmol/l), TC-HDL-cholesterol ratio, and LDL-HDL-cholesterol ratio. Each row provides the mean, standard error of the mean (SEM), minimum (Min), and maximum (Max) values for each characteristic.

Navigate back to Table 1.

## Table 2 Long description

The table presents the macronutrient composition of daily food consumed by overweight adults during three dietary periods: Baseline, MT, HFWL, and HPWL. It includes data on energy intake, fat percentage, protein percentage, carbohydrate percentage, alcohol percentage, fiber percentage, and fiber content in grams. The table has five rows and eight columns. Column headers are Diet, Energy (kcal/d), Fat (percent), Protein (percent), CHO (percent), Alcohol (percent), Fibre (percent), and Fibre (g). Row labels are Baseline, MT, HFWL, HPWL, and P-value. Row 1: Baseline, 2170, 32.9, 17.1, 45.9, 2.09, 2.03, 22.5. Row 2: MT, 2850, 30.2, 15.1, 52.7, 0.16, 1.86, 27.7. Row 3: HFWL, 1880, 35.0, 15.1, 47.1, 0.03, 2.87, 28.2. Row 4: HPWL, 1900, 34.9, 29.9, 33.9, 0.07, 1.32, 13.1. Row 5: P-value, < 0.001, < 0.001, < 0.001, < 0.001, < 0.001, < 0.001, < 0.001.

Navigate back to Table 2.

## Table 3 Long description

The table presents mean anthropometric data of overweight adults after consuming MT, HFWL, and HPWL diets. It includes measurements such as weight, BMI, weight loss, body surface area, SBP, DBP, pulse, waist circumference, hip circumference, and WHR. The table has 10 rows and 6 columns, including a column for the diet type and columns for each measurement. Each row corresponds to a different measurement, and each column corresponds to a different diet or statistical value. Notable trends include variations in weight loss, BMI, and other measurements across the different diets.

Navigate back to Table 3.

## Table 4 Long description

A table comparing body water and three-compartment model results for overweight adults on different diets. The table has 10 rows and 6 columns. Column headers are Diet, MT, HFWL, HPWL, SED, and P. Row labels include H2O volume at 36°C (L), FM (kg), FM (%), FFM (kg), FFM (%), Protein mineral mass (kg), and FFM hydration factor. Row 1: Diet, MT, 48.1, HFWL, 46.6, HPWL, 47.4, SED, 0.40, P, 0.002. Row 2: H2O volume at 36°C (L), FM (kg), MT, 36.6, HFWL, 31.8, HPWL, 31.2, SED, 0.81, P, < 0.001. Row 3: FM (%), MT, 34.9, HFWL, 32.0, HPWL, 31.5, SED, 0.67, P, < 0.001. Row 4: FFM (kg), MT, 66.3, HFWL, 64.3, HPWL, 65.1, SED, 0.39, P, < 0.001. Row 5: FFM (%), MT, 65.1, HFWL, 68.0, HPWL, 68.6, SED, 0.67, P, < 0.001. Row 6: Protein mineral mass (kg), MT, 18.4, HFWL, 18.0, HPWL, 18.0, SED, 0.21, P, 0.081. Row 7: FFM hydration factor, MT, 0.721, HFWL, 0.719, HPWL, 0.722, SED, 0.003, P, 0.608.

Navigate back to Table 4.

## Table 5 Long description

A table comparing predicted RMR, meal energy, and macronutrient content across different test meals. The table has 12 rows and 6 columns. Column headers are Test meal, MT, HFWL, HPWL, SED, and P. Row labels include Predicted RMR (kcal/d), 100 percent RMR (KJ/d), 100 percent RMR (kcal/d), Meal Energy (kJ), Fat (g), Fat (kJ), Fat (percent Energy), Protein (g), Protein (kJ), Protein (percent Energy), CHO (g), CHO (kJ), CHO (percent Energy), Fibre (g), Fibre (kJ), Fibre (percent Energy), Dinner (evening before test day) (kJ), and TEF (kJ). Row 1: Test meal, MT, HFWL, HPWL, SED, P. Row 2: Predicted RMR (kcal/d), 2020, 1890, 1910, 16, < 0.001. Row 3: 100 percent RMR (KJ/d), 8690, 8070, 7980, 219, < 0.001. Row 4: 100 percent RMR (kcal/d), 2080, 1930, 1900, 52, < 0.001. Row 5: Meal Energy (kJ), 4010, 3570, 3530, 103, < 0.001. Row 6: Fat (g), 32.2, 33.3, 33.7, 1.00, 0.29. Row 7: Fat (kJ), 1190, 1230, 1250, 37, 0.29. Row 8: Fat (percent Energy), 29.9, 34.9, 35.4, 0.20, < 0.001. Row 9: Protein (g), 34.8, 31.1, 62.5, 1.66, < 0.001. Row 10: Protein (kJ), 591, 529, 1060, 28, < 0.001. Row 11: Protein (percent Energy), 14.9, 15.0, 30.1, 0.39, < 0.001. Row 12: CHO (g), 134, 104, 73, 3.18, < 0.001. Row 13: CHO (kJ), 2140, 1670, 1180, 51, < 0.001. Row 14: CHO (percent Energy), 53.8, 47.3, 33.2, 0.42, < 0.001. Row 15: Fibre (g), 6.91, 12.2, 6.10, 0.38, < 0.001. Row 16: Fibre (kJ), 55, 98, 49, 3, < 0.001. Row 17: Fibre (percent Energy), 1.40, 2.78, 1.38, 0.03, < 0.001. Row 18: Dinner (evening before test day) (kJ), 3970, 1710, 1600, 140, < 0.001. Row 19: TEF (kJ), 381, 284, 355, 29, 0.006.

Navigate back to Table 5.

## Table 6 Long description

The table presents data on lipid and glucose results of overweight fasted adults (time 0) and after consumption of MT, HFWL, and HPWL meals on the test day. It includes measurements at different time points (0, 180, and 360 minutes) for various parameters such as cholesterol, HDL-cholesterol, LDL-cholesterol, TC-HDL-cholesterol ratio, LDL-HDL-cholesterol ratio, NEFA, TAG, glucose, insulin, HOMA-IR, HOMA-βcell, and IGR. The table has 13 rows and 10 columns. Column headers include Time (min), MT, HFWL, HPWL, SEDmeal, SEDtime.meal, Pmeal, and Ptime.meal. Row labels include different parameters measured. Each row provides values for these parameters at different time points and under different diet conditions. Notable trends include significant reductions in various lipid and glucose parameters after the HFWL and HPWL diets compared to the baseline (MT) diet, with no significant differences between the HFWL and HPWL diets.

Navigate back to Table 6.

## Table 7 Long description

A table with four rows and seven columns comparing different diet parameters and their effects on excretion times. The columns are labeled Diet, t lag (min), t 0.5 (min), t lat (min), and t asc (min). The rows are labeled Mean, MT, HFWL, HPWL, SED, P diet, and P contrasts. Each row provides specific values for each diet type and statistical measures. The table shows mean values and standard errors for different diets, along with p-values indicating the significance of differences between diets.

Navigate back to Table 7.
