# Supplementary figures and images for: Big breakfast diet composition impacts on appetite control and gut health: a randomised weight loss trial in adults with overweight or obesity
Source: Br J Nutr. 2026 Feb 11;135(11):1258–72. doi: 10.1017/S000711452610645X (PMC13423525; doi:10.1017/S000711452610645X)

## Slide 1
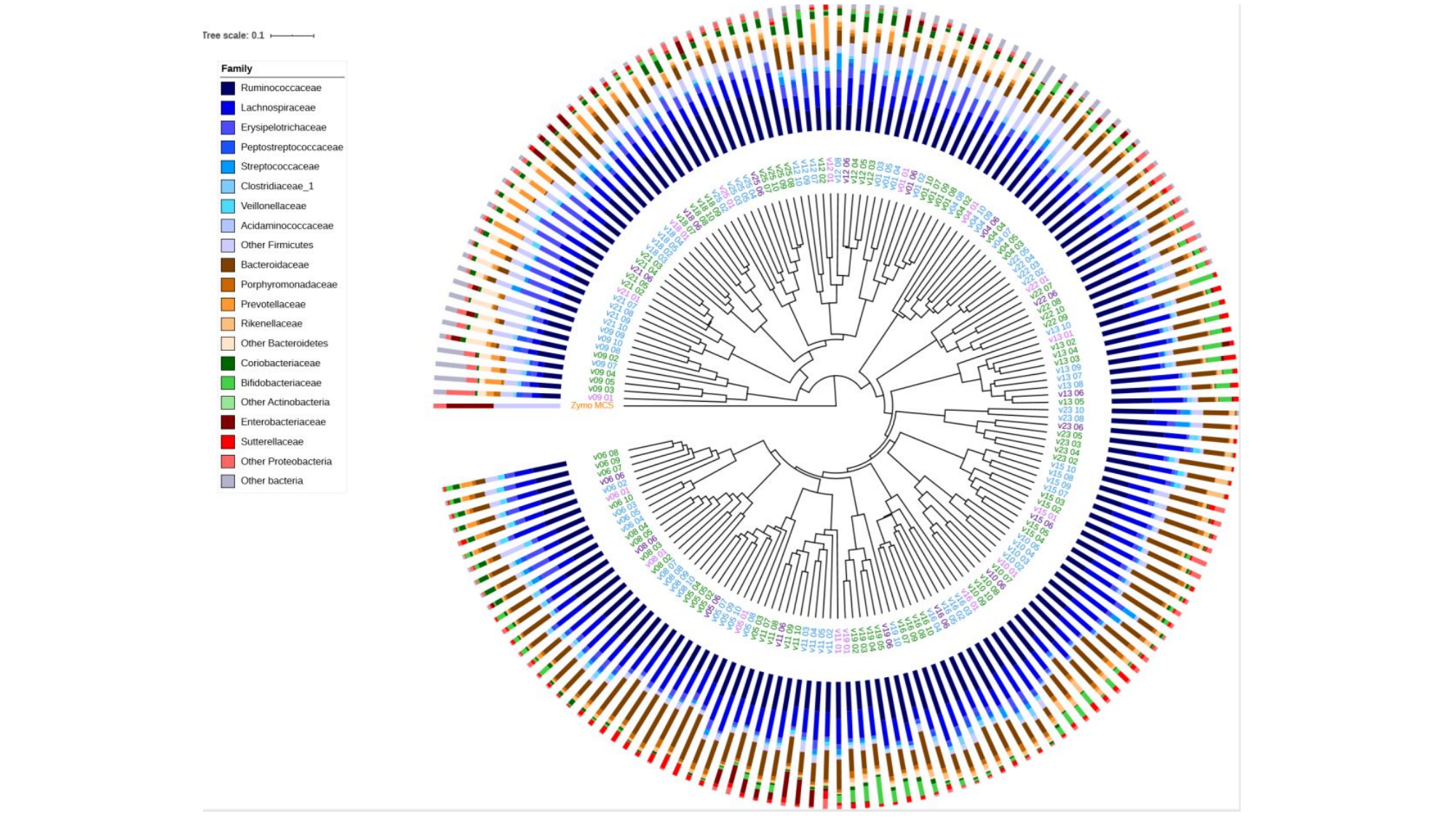

Supplement: Fyfe et al. supplementary material 1 — Fyfe et al. supplementary material [file S000711452610645Xsup001.pptx]

## Slide 1
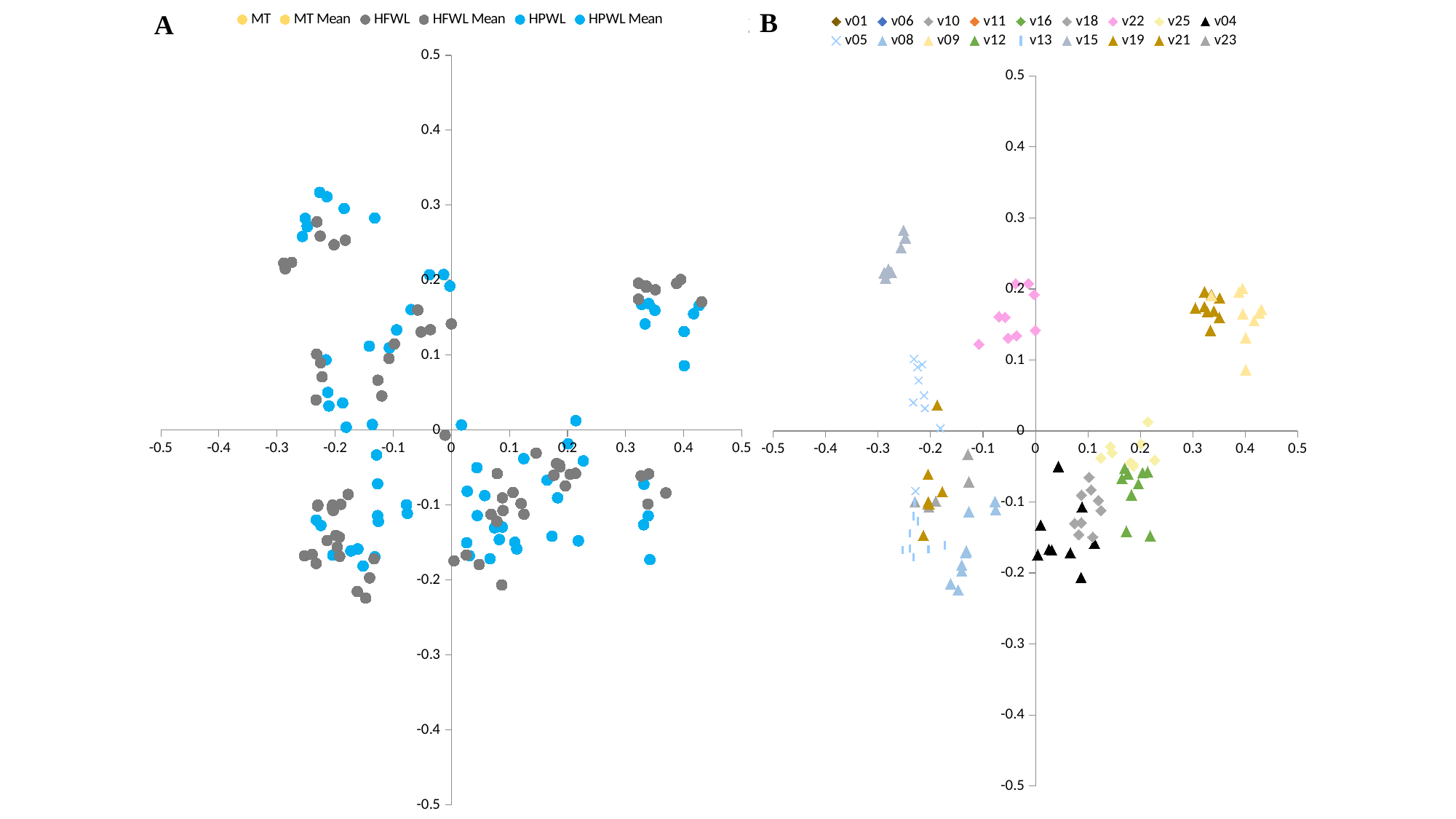

### Chart
| Category | | | | | | |
|---|---|---|---|---|---|---|B
2B
### Chart
| Category | | | | | | | | | | | | | | | | | | |
|---|---|---|---|---|---|---|---|---|---|---|---|---|---|---|---|---|---|---|A

Supplement: Fyfe et al. supplementary material 2 — Fyfe et al. supplementary material [file S000711452610645Xsup002.pptx]

## Slide 1
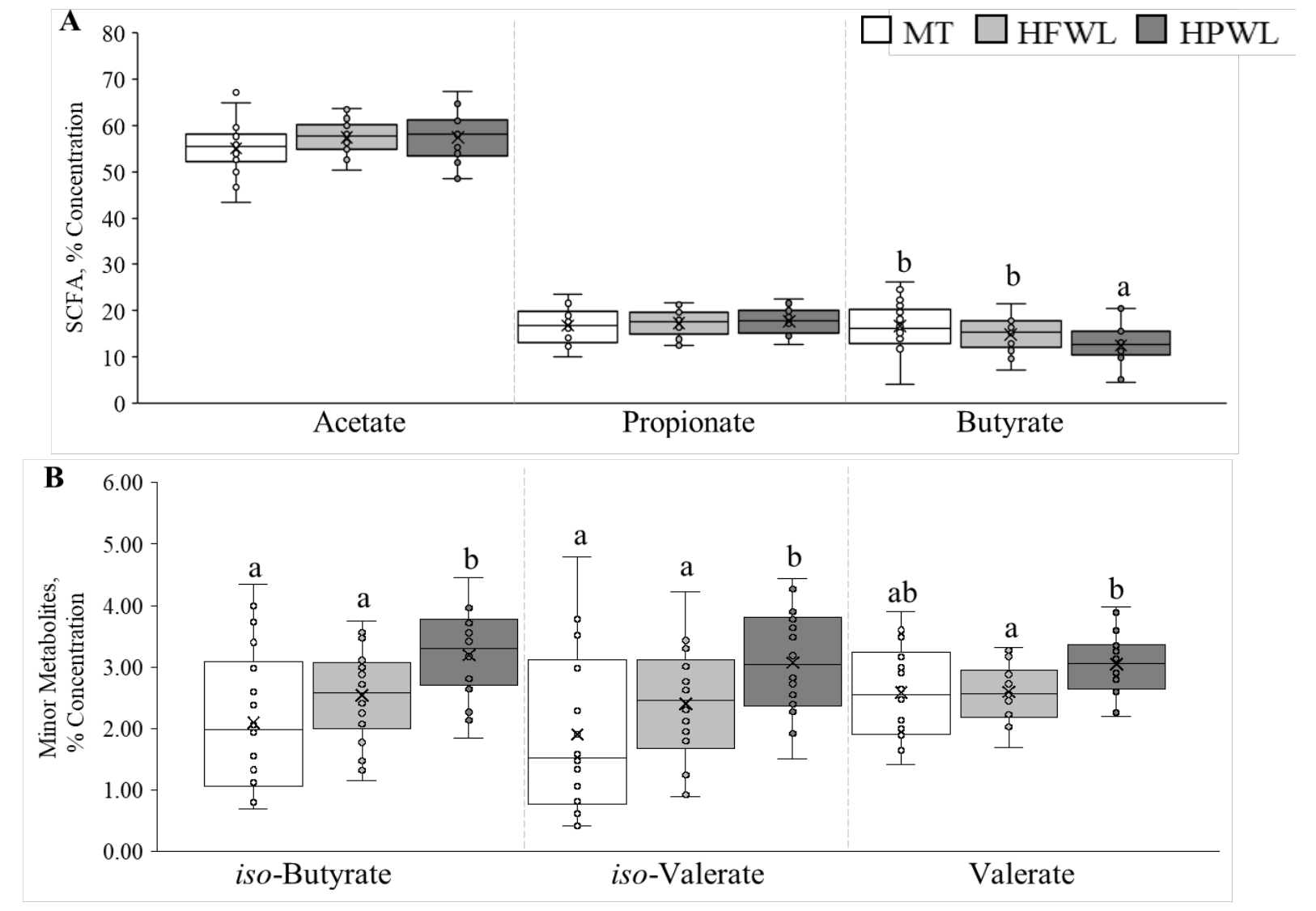

Supplement: Fyfe et al. supplementary material 3 — Fyfe et al. supplementary material [file S000711452610645Xsup003.pptx]
