# Supplementary material for: Big breakfast diet composition impacts on appetite control and gut health: a randomised weight loss trial in adults with overweight or obesity
Source: Br J Nutr. 2026 Feb 11;135(11):1258–72. doi: 10.1017/S000711452610645X (PMC13423525; doi:10.1017/S000711452610645X)
Supplement: Fyfe et al. supplementary material 4 — Fyfe et al. supplementary material [file S000711452610645Xsup004.docx]

**Big breakfast diet composition impacts on appetite control and gut health: a randomized weight loss trial in adults with overweight or obesity.**

**Fyfe, C *et al*.**

**Online Supplementary Material: Technical Annex – Methods**

**Gut hormones**

Human Total Ghrelin kit manufactured by EMD Millipore Corporation, St Louis, Missouri 63103, USA (product code #EZGRT-89K, #EZGRA-89BK).  The assay principle is a sandwich ELISA. Ghrelin (both active and des-octanoyl forms) in the sample forms a complex with reagent anti-human ghrelin IgG (capture antibody). This complex binds to anchor antibodies immobilized on a microtiter plate. A second biotinylated antibody to ghrelin simultaneously binds to the complex. After a wash step a horseradish peroxidase conjugate is added to the immobilized biotinylated antibodies. Following a second wash step addition of substrate 3, 3’, 5, 5’-tetra-methylbenzidine (TMB) and subsequent acidification allows enzyme activity to be measured spectrophotometrically by measuring the increase in absorbance at 450nm. The absorbance (and therefore the enzyme activity) is directly proportional to the total ghrelin concentration.  Controls are provided lyophilized as part of the kit requiring reconstituting in 500µL of deionized water.  A reference standard is provided lyophilized as part of the kit. The standard is reconstituted in 2 mL deionized water.  Between batch imprecision; 9.6% at 317 pg/mL and 3.2% at 1831 pg/mL (in-house data).

The Human Total GIP kit is manufactured by MesoScale Discovery (MSD) Rockville, MD, USA (product code K151RPD1).  Total GIP is measured by sandwich immunoassay. Sample binds to a monoclonal anti-GIP coated MSD plate and incubated for 2 hours. After washing, a solution containing rabbit polyclonal anti-GIP labelled with an electrochemiluminescent compound, MSD SULFO-TAG is added to the plate. The labelled detection antibody binds to the bound GIP to complete the sandwich. After washing, MSD read buffer is added to the plate. This provides the appropriate chemical environment for electrochemiluminescence. The plate is loaded into MSD s600 instrument for analysis. Inside the MSD instrument, a voltage applied to the plate electrodes which causes the labels bound to the electrode surface to emit light. The instrument measures intensity of emitted light to afford a quantitative measure of total GIP present in the sample. Results are calculated using MSD Workbench software. All reagents and standards are supplied by MSD.  A solution of synthetic human GIP protein (residues 1-42) is used to calibrate the assay.  Between batch imprecision; 14.3% at 13.9 pg/mL, 10.4% at 43.5 pg/mL & 11.8% at 154.9 pg/mL (in-house data).

The Total GLP-1 kit is manufactured by MesoScale Discovery (MSD) Rockville, MD, USA (product code K150JVC).  The Total GLP-1 (ver. 2) Assay detects all isoforms of GLP-1 in a sandwich immunoassay. Total GLP-1 & Active GLP-1 capture antibodies are bound to an MSD assay plate. Sample is added to the plate and incubated for 2 hours. GLP-1 in the sample binds to capture antibodies immobilized on the working electrode surface. After washing, a solution containing anti-GLP-1 labelled with an electrochemiluminescent compound, MSD SULFO-TAG is added to the plate. The labelled detection antibody binds to the bound GLP-1 to complete the sandwich. After washing, MSD read buffer is added to the plate. This provides the appropriate chemical environment for electrochemiluminescence. The plate is loaded into MSD s600 instrument for analysis. Inside the MSD instrument, a voltage applied to the plate electrodes which causes the labels bound to the electrode surface to emit light. The instrument measures intensity of emitted light to afford a quantitative measure of GLP-1 present in the sample. Results are calculated using MSD Workbench software.  All reagents and standards are supplied by MSD.  A solution of synthetic amidated human GLP-1 (amino acids 7-36) is used to calibrate the assay.  Between batch imprecision; 8.5% at 6.2 pg/mL, 6.0% at 33.9 pg/mL, 6.1% at 97.5 pg/mL & 7.7% at 821.7 pg/mL (in-house data).

The Human Total PYY kit is manufactured by MesoScale Discovery (MSD) Rockville, MD, USA (product code K151MPD1).  Total PYY is measured by sandwich immunoassay. Sample and biotinylated PYY monoclonal antibody are added to a streptavidin-coated MSD plate and incubated for 2 hours. After washing, a solution containing rabbit polyclonal anti-PYY labelled with an electrochemiluminescent compound, MSD SULFO-TAG is added to the plate. The labelled detection antibody binds to the bound PYY to complete the sandwich. After washing, MSD read buffer is added to the plate. This provides the appropriate chemical environment for electrochemiluminescence. The plate is loaded into MSD s600 instrument for analysis. Inside the MSD instrument, a voltage applied to the plate electrodes which causes the labels bound to the electrode surface to emit light. The instrument measures intensity of emitted light to afford a quantitative measure of total PYY present in the sample. Results are calculated using MSD Workbench software.  All reagents and standards are supplied by MSD.  A solution of synthesized human PYY peptide (amino acids 3-36) is used to calibrate the assay.  Between batch imprecision; 9.9% at 65.1 pg/mL. 6.6% at 236.4 pg/mL & 6.1% at 966.1 pg/mL (in-house data).

**Gastric emptying**

Rate of CO_2_ production (*P*, mmol/min) per participant was estimated from body surface area using the height-weight formula defined by Haycock et al^(1)^. ^13^CO_2_ enrichments were expressed as atom percent excess (APE) and were corrected for baseline APE. The rate of appearance of ^13^CO_2_ in the breath (*R,* mmol/min) was calculated as:

$R=\frac{0.01 \text{APE}\left( t_{1} \right)+0.01 \text{APE}\left( t_{0} \right)}{t_{1}-t_{0}} P$, where $t_{0}$ and $t_{1}$are two consecutive time points (min) at which measurements were taken. The total appearance of ^13^CO_2_ in the breath during this time window (*T*, mmol) was calculated as $(t_{1}-t_{0}) R$. The cumulative ^13^CO_2_ appearance in the breath up to time *t* as % of the dose was obtained from summing *T* over all preceding time windows multiplied by 100 divided by dose. It is denoted by *y(t)*. These cumulative data were fitted to the $mk\beta$ model: $y(t)=m \left( 1-\exp\left( -k t \right) \right)^{\beta}$ ^(2)^, using the non-linear regression routine nls in R (R Core Team, 2016).

The model parameters *k* and $\beta$ were converted into biologically meaningful parameters as follows: $t_{0.5}$ is the time point at which 50% of the total excretion of ^13^CO_2_ in the breath has been recovered: $t_{0.5}=-\frac{1}{k} ln(1-2^{-\frac{1}{\beta}})$ (16). $t_{lag}$ is the time point at which the rate of excretion is at its maximum. It also corresponds to the inflection point of the accumulation curve: $t_{lag}=\frac{\ln\left( \beta\right)}{k}$ (15). Schommartz et al^(3)^ divided $t_{0.5}$ into a measure for initial delay (latency) in ^13^CO_2_ excretion (denoted as $t_{lat}$) and a measure for the length of time during which excretion is rapid, i.e. when the cumulative curve is ascending ($t_{asc}$): $t_{lat}=\frac{\ln\left( \beta\right)}{k}+\frac{1}{k} (\frac{1}{\beta}-1)$ and $t_{asc}= -\frac{1}{k} [ \ln\left( 1-2^{-\frac{1}{\beta}} \right)+\ln\left( \beta\right)+\frac{1}{\beta}-1 ]$.

**Fecal Sample Analysis: DNA extraction and quantitative real-time PCR (qPCR)**

**DNA Extraction**

Fresh fecal samples were collected from volunteers and one part feces combined with two parts phosphate buffered saline (containing 30% glycerol) in gentleMACS M Tubes (Cat #130-093-236, Miltenyi Biotec, Germany). A homogenous slurry was produced by processing in a gentleMACS Dissociator (Cat #130-093-235, Miltenyi Biotec, Germany) for a 65 second cycle. 350 μL of the resulting slurry was collected and stored at -70˚C for a maximum of one month before DNA was extracted using the FastDNA™ Spin Kit for Soil (Cat #116560200, MP Biomedicals, Germany); the standard kit protocol was followed for this plus an additional 5 minute incubation stage at 55˚C. DNA was eluted in a volume of 100 μL, then used as the template for two different techniques; preparation of 16S rRNA gene amplicon libraries for Illumina MiSeq sequencing to determine the composition of the bacterial community, and quantification of the 16S rRNA gene by qPCR to determine the bacterial load.

**qPCR**

DNA concentrations were measured using Qubit 2.0 Fluorometer (Thermo Fisher Scientific, Paisley, UK). The total number of 16S rRNA gene copies was determined as described previously^(4)^ with universal primers (UniF, GTGSTGCAYGGYYGTCGTCA; UniR, ACGTCRTCCMCNCCTTCCTC; 500 nM each) and 2 ng DNA in a total volume of 10 µl. Standard curves were produced from a 5-fold dilution series of a 16S rRNA gene amplicon from a reference strain (*Ruminococcus bromii* L2-63). The abundance of 16S rRNA genes was determined from the standard curves and expressed as 16S rRNA gene copies per gram of feces.

**16S rRNA gene amplicon sequencing/Preparation of Illumina MiSeq library**

To prepare the amplicon library, the hypervariable V1-V2 region of the 16S rRNA gene was amplified using primers adapted for Illumina MiSeq. Each DNA sample was amplified with an individually barcoded 338rcbc primer (5’-CAAGCAGAAGACGGCATACGAGAT-barcode-AGTCAGTCAGAAGCTGCCTCCCGTAGGAGT-3´) alongside 27F primer (5’-AATGATACGGCGACCACCGAGATCTACACTATGGTAATTCCAGGTTYGATYMTGGCTCAG-3´) in 25 µl PCR reactions containing both primers at 10 µM. The PCR reactions also contained 5X Q5 Reaction Buffer, Q5 High-Fidelity DNA Polymerase, 10 mM dNTPs (Cat #M0491 and #N0447, New England Biolabs, United States) and Nuclease-free Water as per the Q5 standard protocol. Quadruplicate PCR reactions were run for each sample to minimize potential amplification bias effects. Reactions were run at 98°C for 2 mins, followed by 20 cycles of 98°C for 30 secs, 50°C for 30 secs, 72°C for 90 secs before a final extension of 72°C for 5 mins. Quadruplicate reactions for each sample were then pooled, and products confirmed by running on an agarose gel. Products were purified using ethanol precipitation then quantified with the Qubit dsDNA HS Assay (Cat #Q32854, Invitrogen, United States). The amplicon library was prepared by combining together all products together at the same concentration in an equimolar mastermix, which was then submitted for Illumina MiSeq sequencing (2 x 250 bp read length) by the Centre for Genome-Enabled Biology and Medicine (CGEBM) at the University of Aberdeen. Sequencing primers (Read 1: 5’-GAGATCTACACTATGGTAATT CCAGMGTTYGATYMTGGCTCAG-3´; Read 2: 5’-AGTCAGTCAGAAGCTGCCTCCCGTAGGAGT-3´; Index Sequencing Primer: 5’-ACTCCTACGGGAGGCAGCTTCTGACTGACT-3´) were also supplied (all at 100 μM) for running alongside the library. The concentration of the library was confirmed by qPCR and a final purification step to remove any potential contaminants was carried out by CGEBM using the AMPure XP system (Cat #A63881, Beckman Coulter, United States) prior to sequencing.

**Illumina MiSeq Sequence Data Analysis**

Once run, Illumina MiSeq data was analyzed using mothur software (versions 1.42.3 and 1.39.5) and a modified version of the MiSeq standard operating procedure^(5)^ similar to that described by Dalby *et al*., 2017^(6)^. In brief, contigs were assembled from forward and reverse reads, with only those between 280-470 bases taken forward for analysis. Sequences were aligned against the SILVA reference database and Operational Taxonomic Unit (OTU) generated using OptiClust in mothur^(7)^ at 97% similarity. Taxonomy was assigned to each OTU using the Ribosomal Database Project (RDP) reference database as implemented in mothur. Representative sequences were obtained using the get.otureps command in mothur to allow selective manual curation of taxonomic identifications using Basic Local Alignment Search Tool (BLAST) against the National Center for Biotechnology Information (NCBI) nucleotide reference database^(8)^. No chimera removal software was used, as our benchmarking has shown that this results in artefactual removal of “real” sequences. Instead, all sequences with 10 reads or less across the whole dataset were removed. Samples were then sub-sampled to 4600 reads per sample to allow comparison across all samples. All samples below this threshold were deemed not to have sufficient sequencing depth (*n*=18 volunteers, *n*=173 samples).

Statistical analysis was carried out as described by Dalby et al., 2017^(6)^. α- diversity measures were run within mothur, and outputs plotted using Microsoft Excel with *P* values to determine significance generated using Satterthwaite's method (*P* values <0.05 reported as being significant)^(9)^. Analysis of composition changes shown by β-diversity measures were also run within mothur using AMOVA (Analysis of Molecular Variance) and Parsimony tests (*P* values or ParSig <0.05 reported). Output files were loaded into the Interactive Tree of Life (iTOL) website^(10)^ to generate phylogenetic trees, Principal Coordinates Analysis (PCoA) data generated using mothur was plotted in Microsoft Excel. Specific composition changes at Operational Taxonomic Unit (OTU), Genus, Family and Phylum levels were identified using the LEfSe^(11)^ and Metastats^(12)^ approaches, as implemented in mothur. Metastats *P* values were corrected for multiple comparisons using the Benjamini-Hochberg approach, with only corrected *P* values <0.05 being reported as significant.

The sequence data from this study is available in the European Nucleotide Archive under study accession number ERP121324, with samples registered as sample accessions ERS4531430-ERS4531602 (**Supplementary Table 5**).

**ABBREVIATIONS:** APE, atom percent excess; BLAST, Basic Local Alignment Search Tool; CGEBM, Centre for Genome-Enabled Biology and Medicine; GIP, Glucose-dependent Insulinotropic Polypeptide; GLP-1, Glucagon-Like Peptide-1; iTOL, Interactive Tree of Life; MSD, MesoScale Discovery; NCBI, National Center for Biotechnology Information; OTU, Operational Taxonomic Unit; PYY, Peptide YY; RDP, Ribosomal Database Project; t_0.5_, timepoint at which 50% of the total excretion of ^13^CO_2_ in the breath has been recovered; t_asc_, the length of time during which ^13^CO_2_ excretion in the breath is rapid i.e. when the cumulative curve is ascending; t_lag_, timepoint at which ^13^CO_2_ excretion rate in the breath is at its maximum; t_lat_, initial delay or latency of ^13^CO_2_ excretion in the breath

**REFERENCES:**

1. Haycock GB, Schwartz GJ & Wisotsky DH. (1978) Geometric method for measuring body surface area: a height-weight formula validated in infants, children, and adults. *J Pediatr* **93**, 62-6. doi: 10.1016/s0022-3476(78)80601-5
2. Ghoos YF, Maes BD, Geypens BJ *et al*. (1993) Measurement of gastric emptying rate of solids by means of a carbon-labeled octanoic acid breath test. *Gastroenterology* **104**, 1640-1647. doi: 10.1016/0016-5085(93)90640-x
3. Schommartz B, Ziegler D & Schadewaldt P. (1998) Significance of diagnostic parameters in [13C] octanoic acid gastric emptying breath tests. *Isotopes Environ Health Stud* **34**, 135-143. PMID: 9854848
4. Reichardt N, Vollmer M, Holtrop G *et al*. (2018) Specific substrate-driven changes in human faecal microbiota composition contrast with functional redundancy in short-chain fatty acid production. *ISME J* **12**, 610-22.
5. Kozich JJ, Westcott SL, Baxter NT *et al*. (2013) Development of a dual-index sequencing strategy and curation pipeline for analyzing amplicon sequence data on the MiSeq Illumina sequencing platform. *Appl Environ Microbiol* **79**, 5112-20. doi: 10.1128/AEM.01043-13
6. Dalby MJ, Ross AW, Walker AW *et al*. (2017) Dietary Uncoupling of Gut Microbiota and Energy Harvesting from Obesity and Glucose Tolerance in Mice. *Cell Rep* **21**, 1521-1533. doi: 10.1016/j.celrep.2017.10.056
7. Westcott SL & Schloss PD. (2017) OptiClust, an Improved Method for Assigning Amplicon-Based Sequence Data to Operational Taxonomic Units. *mSphere* **2**, e00073-17. doi: 10.1128/mSphereDirect.00073-17
8. Camacho C, Coulouris G, Avagyan V *et al*. (2009) BLAST+: architecture and applications. *BMC Bioinformatics* **10**, 421. doi: 10.1186/1471-2105-10-421
9. Satterthwaite, FE. (1946) An approximate distribution of estimates of variance components. *Biometrics* **2**, 110-114. PMID: 20287815
10. Letunic I & Bork P. (2024) Interactive Tree of Life (iTOL) v6: recent updates to the phylogenetic tree display and annotation tool. *Nucleic Acids Res* **52**, W78-82. doi: 10.1093/nar/gkae268
11. Segata N, Izard J, Waldron L *et al*. (2011) Metagenomic biomarker discovery and explanation. *Genome Biol* **12**, R60. doi: 10.1186/gb-2011-12-6-r60
12. White JR, Nagarajan N & Pop M. (2009) Statistical methods for detecting differentially abundant features in clinical metagenomic samples. *PLoS Comput Biol* **5**, e1000352. doi: 10.1371/journal.pcbi.1000352
