# Supplementary material for: Big breakfast diet composition impacts on appetite control and gut health: a randomised weight loss trial in adults with overweight or obesity
Source: Br J Nutr. 2026 Feb 11;135(11):1258–72. doi: 10.1017/S000711452610645X (PMC13423525; doi:10.1017/S000711452610645X)
Supplement: Fyfe et al. supplementary material 5 — Fyfe et al. supplementary material [file S000711452610645Xsup005.docx]

Online Supplementary Material Table 1: CONSORT 2010 checklist of information to include when reporting a randomized trial

| Section/Topic | Item No | Checklist item | Reported on page No |
| --- | --- | --- | --- |
| Title and abstract | | | |
|  | 1a | Identification as a randomized trial in the title | 1 |
|  | 1b | Structured summary of trial design, methods, results, and conclusions | 3 |
| Introduction | | | |
| Background and objectives | 2a | Scientific background and explanation of rationale | 4-5 |
|  | 2b | Specific objectives or hypotheses | 5 |
| Methods | | | |
| Trial design | 3a | Description of trial design (such as parallel, factorial) including allocation ratio | 5-6 |
|  | 3b | Important changes to methods after trial commencement (such as eligibility criteria), with reasons | *N/A* |
| Participants | 4a | Eligibility criteria for participants | 5-6 |
|  | 4b | Settings and locations where the data were collected | 5-6 |
| Interventions | 5 | The interventions for each group with sufficient details to allow replication, including how and when they were actually administered | 5-13 |
| Outcomes | 6a | Completely defined pre-specified primary & secondary outcome measures, including how & when they were assessed | 3, 5-13 |
|  | 6b | Any changes to trial outcomes after the trial commenced, with reasons | *N/A* |
| Sample size | 7a | How sample size was determined | 13 |
|  | 7b | When applicable, explanation of any interim analyses and stopping guidelines | *N/A* |
| **Randomization** | | | |
| Sequence generation | 8a | Method used to generate the random allocation sequence | 12 |
|  | 8b | Type of randomization; details of any restriction (such as blocking and block size) | 12 |
| Allocation concealment mechanism | 9 | Mechanism used to implement the random allocation sequence (such as sequentially numbered containers), describing any steps taken to conceal the sequence until interventions were assigned | *N/A* |
| Implementation | 10 | Who generated the random allocation sequence, who enrolled participants, and who assigned participants to interventions | 2 |
| Blinding | 11a | If done, who was blinded after assignment to interventions (for example, participants, care providers, those assessing outcomes) and how | *N/A* |
|  | 11b | If relevant, description of the similarity of interventions | *N/A* |
| Statistical methods | 12a | Statistical methods used to compare groups for primary and secondary outcomes | 12-13 |
|  | 12b | Methods for additional analyses, such as subgroup analyses and adjusted analyses | 12-13 |
| \| Section/Topic \| Item No \| Checklist item \| Reported on page No \| \| --- \| --- \| --- \| --- \| | | | |
| Results | | | |
| Participant flow | 13a | For each group, the numbers of participants who were randomly assigned, received intended treatment, and were analysed for the primary outcome | 5-6, 42, Fig. 1 |
|  | 13b | For each group, losses and exclusions after randomization, together with reasons | 5-6, 42, Fig. 1 |
| Recruitment | 14a | Dates defining the periods of recruitment and follow-up | 6 |
|  | 14b | Why the trial ended or was stopped | *N/A* |
| Baseline data | 15 | A table showing baseline demographic and clinical characteristics for each group | 35 |
| Numbers analysed | 16 | For each group, number of participants (denominator) included in each analysis and whether the analysis was by original assigned groups | 6, 13, 35-44 |
| Outcomes and estimation | 17a | For each primary and secondary outcome, results for each group, and the estimated effect size and its precision (such as 95% confidence interval) | 13-20, 35-44 |
|  | 17b | For binary outcomes, presentation of both absolute and relative effect sizes is recommended | *N/A* |
| Ancillary analyses | 18 | Results of any other analyses performed, including subgroup analyses and adjusted analyses, distinguishing pre-specified from exploratory | 13-20, 35-44 |
| Harms | 19 | All important harms or unintended effects in each group (for specific guidance see CONSORT for harms) | *N/A* |
| Discussion | | | |
| Limitations | 20 | Trial limitations, addressing sources of potential bias, imprecision, and, if relevant, multiplicity of analyses | 24 |
| Generalizability | 21 | Generalizability (external validity, applicability) of the trial findings | 20-25 |
| Interpretation | 22 | Interpretation consistent with results, balancing benefits and harms, and considering other relevant evidence | 20-25 |
| Other information | | |  |
| Registration | 23 | Registration number and name of trial registry | 2, 6 |
| Protocol | 24 | Where the full trial protocol can be accessed, if available | *N/A* |
| Funding | 25 | Sources of funding and other support (such as supply of drugs), role of funders | 1, 26 |

Abbreviations: CONSORT, Consolidated Standards of Reporting Trials; N/A, not applicable
