# Supplementary material for: Big breakfast diet composition impacts on appetite control and gut health: a randomised weight loss trial in adults with overweight or obesity
Source: Br J Nutr. 2026 Feb 11;135(11):1258–72. doi: 10.1017/S000711452610645X (PMC13423525; doi:10.1017/S000711452610645X)
Supplement: Fyfe et al. supplementary material 6 — Fyfe et al. supplementary material [file S000711452610645Xsup006.docx]

**Online Supplementary Material Table 2: Menu for the 3d MT and 7d Washout dietary periods^1^**

| **Menu day** | **Study days** | **Breakfast** | **Lunch** | **Dinner** |
| --- | --- | --- | --- | --- |
| 1  (& Test Day B*) | 8, 43 | Omelette, Potato Waffle, Baked Beans,  Toast & Orange Juice | Chicken Mayo Salad with Pitta Bread,  Chocolate Marshmallow Crispy Cake | Beef Stroganoff with Rice,  Banoffee Pie |
| 2 | 5, 40 | Alpen & Milk, Crumpets & Cheese,  Fruit Yoghurt | Bacon, Lettuce & Tomato Roll,  Apple & Apricot Muffin, Apple Juice | Pasta Bolognaise with Garlic Bread,  Steamed Chocolate Pudding  Tinned Peaches |
| 3  (& Test Day A^†^) | 6, 41 | Special K & Milk, Cheese & Ham Toasted Sandwich, Fruit Yoghurt, Apple Juice | Chicken Mayo Salad, Potato Crisps,  Orange Juice, Fruit Muffin & Jam | Beef Stir Fry with Noodles,  Lemon Drizzle Pudding |
| 4 | 7, 42 | Corn Flakes & Milk, Fruit Smoothie,  Toast & Jam | Cheese & Ham Salad Roll, Apple Juice,  Grapes, Flapjack | Pulled Pork with Sweet Potato Wedges,  Raspberry, Apple & Pear Pudding |
| 5 | 39 | Special K & Milk,  Bacon, Egg, Potato Waffle, Baked Beans,  Toast & Orange Juice | Moroccan Chicken with Cous Cous,  Hummus with Crudités & Pitta Bread,  Chocolate Marshmallow Crispy Cake | Chicken & Pepperoni Pizza,  Toffee Sponge Pudding |
| 6 | 37 | Alpen & Milk, Crumpets & Cheese,  Fruit Yoghurt | Tuna & Sweetcorn Salad Wrap,  Lemon & Coconut Slice | Chicken Curry with Rice,  Chocolate & Banana Pudding |
| 7 | 38 | Corn Flakes & Milk, Fruit Smoothie,  Toast & Jam | Mexican Turkey Pitta Bread & Salad,  Chocolate Orange Slice, Apple Juice | Fish Cakes with Mashed Potato,  Orange Juice,  Victoria Sponge Cake |

Abbreviations: MT, Maintenance diet

* (only menu days 1-4 provided during MT diet; study days 5-8, full 7d menu provided during washout period; study days 37-43)

† Test Day A was day 6 of study, Test Day B was day 8 of study
