# Supplementary material for: Big breakfast diet composition impacts on appetite control and gut health: a randomised weight loss trial in adults with overweight or obesity
Source: Br J Nutr. 2026 Feb 11;135(11):1258–72. doi: 10.1017/S000711452610645X (PMC13423525; doi:10.1017/S000711452610645X)
Supplement: Fyfe et al. supplementary material 7 — Fyfe et al. supplementary material [file S000711452610645Xsup007.docx]

**Online Supplementary Table 3. Menu for the HFWL Diet (7 day rotation, randomized to either study days 9-36 or 44-71)**

| **Menu Day** | **Study days** | **Breakfast** | **Lunch** | **Dinner** |
| --- | --- | --- | --- | --- |
| 1 | 11, 18, 25, 32  or  46, 53, 60, 67 | Shreddies & Milk,  Scrambled Egg on Toast, Orange Juice  Pain au Chocolat | Hummus with Crudités, Grapes, Cereal Bar,  Fruit Yoghurt, Rice & Potato Chip sticks | Chorizo Barley Risotto,  Baguette Slice,  Jelly Candies, Water |
| 2 | 12, 19, 26, 33  or  47, 54, 61, 68 | Special K, Bran Flakes, Chia Seeds & Milk,  Cheese & Tomato Toasted Sandwich,  Banana, Orange Juice | Vegetable & Barley Broth with Wholemeal Roll,  High Fiber Crackers with Cream Cheese,  Peanuts, Fruit Yoghurt, Shortbread Biscuit | Beef Chilli with Rice,  Water |
| 3  (& Test Day A*) | 13, 20, 27, 34  or  48, 55, 62, 69 | Alpen, Sunflower Seeds, Raisins & Milk,  Toasted Bacon Sandwich, Water | Barbecue Chicken & Sweet Potato Salad,  Sandwich Thin, Digestive Biscuit, Apple  Water | Chicken Curry with Brown Rice,  Tinned Peaches |
| 4 | 14, 21, 28, 35  or  49, 56, 63, 70 | Porridge with Mixed Seeds,  Zucchini, Cheese & Walnut Muffin, Strawberries, Orange Juice | Chicken with Vaal Dahl, Roasted Peppers,  Pitta Bread, Banana, Chocolate Biscuit  Quavers Chips, Water | Vegetable Pizza |
| 5 | 15, 22, 29  or  50, 57, 64 | Corn Flakes, Bran Flakes & Milk,  Fried Egg, Sausage, Baked Beans & Toast,  Raspberries, Water | Prawn Noodle Salad, Bread & Butter,  Chocolate & Cereal Biscuits, Apple Juice | Breaded Cod Fillet, New Potatoes & Roasted Mediterranean Vegetables,  Digestive Biscuit, Water |
| 6 | 9, 16, 23, 30  or  44, 51, 58, 65 | Broad Bean Frittata, Sandwich Thin,  Fruit Smoothie,  Apple & Apricot Muffin, Water | Lentil Soup, Egg Mayonnaise Sandwich,  Potato Crisps, Roasted Fava Beans, Pineapple, Water | Bacon & Artichoke Carbonara  Wholemeal Bread, Water |
| 7 | 10, 17, 24, 31  or  45, 52, 59, 66 | Porridge with Raisins,  Beans on Toast, Fruit Yoghurt, Milk | Tomato Soup, Bread & Butter,  Cheese & Ham Sandwich, Roasted Peas,  Shortbread Biscuit, Melon, Water | Goats Cheese, Spinach & Butternut Squash Bake  Custard Cream Biscuit |
| Test Day B* | 36 or 71 | Omelette, Baked Fava Beans,  Toast & Jelly, Orange Juice | Lentil Soup, Egg Mayonnaise Sandwich,  Potato Crisps, Roasted Fava Beans, Pineapple, Water | Bacon & Artichoke Carbonara  Wholemeal Bread, Water |

Abbreviations: HFWL, High Fiber Weight Loss Diet

* Test Day A was day 34 or 69 of study, Test Day B was day 36 or 71 of study (not included in the 7 day rotation)
