# Supplementary material for: Big breakfast diet composition impacts on appetite control and gut health: a randomised weight loss trial in adults with overweight or obesity
Source: Br J Nutr. 2026 Feb 11;135(11):1258–72. doi: 10.1017/S000711452610645X (PMC13423525; doi:10.1017/S000711452610645X)
Supplement: Fyfe et al. supplementary material 8 — Fyfe et al. supplementary material [file S000711452610645Xsup008.docx]

**Online Supplementary Material Table 4: Menu for the HPWL Diet (7 day rotation, randomized to either days 9-36 or 44-71)**

| **Menu Day** | **Study Days** | **Breakfast** | **Lunch** | **Dinner** |
| --- | --- | --- | --- | --- |
| 1 | 11, 18, 25, 32  or  46, 53, 60, 67 | Special K & Milk, Orange Juice,  Scrambled Egg, Bacon, Chicken, Mushrooms & Potato Waffle | Tuna Salad with Pitta Bread,  Lemon & Coconut Slice | Beef Stroganoff with Rice |
| 2 | 12, 19, 26, 33  or  47, 54, 61, 68 | Special K & Milk, Buttered Crumpets,  Turkey Rashers & Boiled Egg,  Fruit Smoothie, Water | Chicken & Bacon Mayonnaise Roll with Salad,  Orange Juice,  Apple & Apricot Muffin | Chicken Curry with Rice |
| 3  (& Test Day A*) | 13, 20, 27, 34  or  48, 55, 62, 69 | Sausage, Bacon, Chicken, Chutney,  Toast & Butter, Fruit Smoothie, Orange Juice | Sweet Chilli Chicken Sandwich with Salad,  Milk, Chocolate Crispy Cake | Mince, Potatoes & Green Beans,  Mandarin Fool |
| 4 | 14, 21, 28, 35  or  49, 56, 63, 70 | Corn Flakes & Milk, Fruit Yoghurt,  Cheese & Ham Toasted Sandwich, Water | Roast Beef Sandwich with Salad,  Blackcurrant Fool | Chicken & Vegetable Pasta |
| 5 | 15, 22, 29  or  50, 57, 64 | Burger, Chicken, Hash Browns, Tomato, Mushrooms, Chutney, Orange Juice,  Toast & Jelly, Milk | Pork Sandwich, Chicken & Vegetable Soup,  Granola | Turkey Chilli with Rice |
| 6 | 9, 16, 23, 30  or  44, 51, 58, 65 | Special K & Milk, Buttered Crumpets,  Turkey Rashers & Boiled Egg,  Fruit Smoothie, Water | Chicken & Gammon Mayonnaise Salad,  Orange Juice,  Raspberry, Apple & Pear Pudding with Cream | Sweet & Sour Beef Stir Fry with Noodles |
| 7 | 10, 17, 24, 31  or  45, 52, 59, 66 | Corn Flakes & Milk, Fruit Yoghurt,  Cheese & Ham Toasted Sandwich, Water | Chicken Mayo Salad with Pitta Bread,  Milk, Digestive Biscuit, Milk Chocolate, Water | Pork Steak, Mashed Potato, Peas, Carrots, Onions in Gravy |
| Test Day B* | 36 or 71 | Ham & Mushroom Omelette, Chutney,  Toast, Milk | Chicken Mayo Salad with Pitta Bread,  Milk, Digestive Biscuit, Milk Chocolate, Water | Beef Stroganoff with Rice |

Abbreviations: HPWL, High Protein Weight Loss Diet

* Test Day A was day 34 or 69 of study, Test Day B was day 36 or 71 of study (not included in the 7 day rotation)
